# A strategy for effective latent HIV reactivation using subtherapeutic drug doses

**DOI:** 10.1038/s41598-017-00097-9

**Published:** 2017-11-30

**Authors:** James Cotterell, G. Gregory Neely

**Affiliations:** 1 The Garvan Institute for Medical Research, 384 Victoria Street, Darlinghurst, Sydney, NSW 2010 Australia; 2 The Dr. John and Anne Chong Lab for Functional Genomics, Charles Perkins Centre and School of Life & Environmental Sciences, The University of Sydney, Camperdown, NSW 2006 Australia

## Abstract

Cell state switches underlie a plethora of biological phenomena and disease treatment strategies. Hence the ability to efficiently switch states in a chosen direction is of central importance in a number of scenarios. Increasing the concentration of an effector that results in a given switch is often limited by side effects. Approaches are thus increasingly sought to bypass these constraints, increasing the frequency of state switching without increasing the frequency of the side effect. Here, we employ dynamical systems theory to uncover a simple strategy as to how to maximize the probability of reactivating latent Human immunodeficiency virus (HIV) whilst maintaining minimal side effects. We demonstrate that continuous supply of an effector is significantly more likely to result in a switch with minimal side effects than the same effector supplied in temporally discrete doses. Importantly this continual dosage is likely to occur far below the Minimum effective dose at a concentration that has classically been thought subtherapeutic. We therefore suggest that in many interventional settings there exists potential to reduce drug dose much further than has previously been thought possible yet still maintaining efficacy.

## Introduction

**Figure 1:**
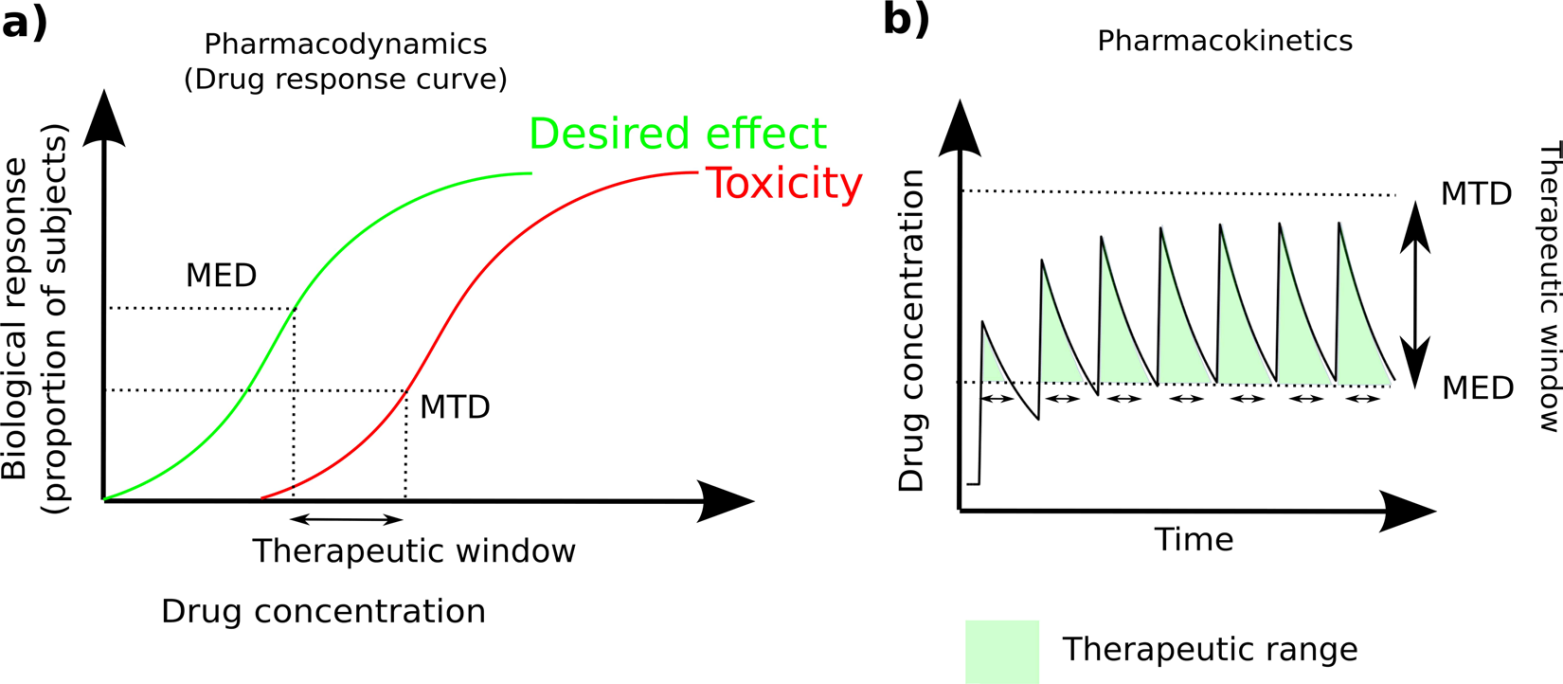
The classic view of pharmacodynamics and pharmacokinetics. (**a**) Illustration of typical dose response curves for desired and side effects. The x-axis shows the drug concentration and the y-axis shows the biological effect. The desired biological effect is shown by the green line and detrimental side effects by the red line. The Minimum effective dose (MED) is defined as the dose above which sufficient desired effect is being achieved. The maximal tolerable dose (MTD) is defined as the dose above which we are causing intolerable side effects. The dosage gap between the MED and MTD is termed the therapeutic window. (**b**) Illustration of a dose scheduling pharmacokinetic profile where one tries to maintain a drug concentration within the therapeutic window. Time is now on the x-axis and drug concentration on the y-axis. The unbroken line specifies the drug concentration and multiple applications of the drug are demonstrated by the multiple peaks which decay with time. The therapeutic window is illustrated by the dashed MED and MTD lines. The green shaded area is drug application that is within the therapeutic window.

Robust control of cell state switches is a primary goal in many fields of biology and medicine. Specific examples of medicinal value include cellular reprogramming and reducing patient viral load via ART in HIV treatment^1,2^. Strategies to efficiently promote switches in a chosen direction are thus highly sought after and have wide applicability. Often an effector can be used that promotes a transition in one particular direction, for example various growth factors can be used to channel cells down particular developmental trajectories whilst drugs are used for medicinal state switching^3^. However, increasing the frequency of transition by increasing the concentration of effector is often limited by other detrimental side effects caused by the effector. In drug development this scenario can be envisaged in a typical pharmacodynamic dose-response graph where greater biological response is seen with increasing dose of the drug^4^ (Fig. 1a). Whilst at lower doses a desired response is achieved, increasing the dose inevitably increases the side effect response. Minimum effective dose (MED) is defined as the drug concentration that results in the minimal sufficient desired response whilst Maximum tolerable dose (MTD) is defined as the dose that results in the maximal amount of side effects that can be tolerated. A common aim in a clinical setting then is to maintain the dose of a drug within a therapeutic window defined by the limits of the MED and MTD which is often achieved by applying multiple doses of the drug at intermittent time points (Fig. 1b).

A prime example of such drug concentration (dose) constraints involves proviral reactivation strategies for curing HIV. Despite the success of combination antiretroviral therapy, it is not currently a cure for HIV since the HIV-1 virus is not totally eradicated. HIV can enter a long lived proviral latent state that is proving to be a significant barrier to cure^5^. Reactivation of latent HIV within CD4+ T cells is thus one of the leading strategies aimed at curing this disease^6^. The aim of reaction strategies is to purge the proviral HIV reservoir and thus flush the remaining virus out. An example of this ‘shock and kill’ style strategy involves proviral reactivation in combination with elimination of viral producing cells using immune effectors^7^. However, to date attempts to reactivate the virus have met with limited success^8^.

These problems have motivated work to explore novel strategies to increase the frequency of HIV reactivation. For example, recently it has been demonstrated how the synergistic addition of noise enhancing substances to proviral activating drugs increases the likelihood of HIV reactivation^9^. It was demonstrated how one mechanism of noise generation leads to a range extension of Long terminal repeats (LTR) activation increasing the likelihood that it will pass a given threshold required for state switching. However, the question remains whether there are other strategies by which one can increase state switching without using higher levels of drug? To answer this question, we explored HIV reactivation using an abstract dynamical systems theory framework. Through such an analysis we elucidate an effective general strategy for increasing the likelihood, not only of HIV reactivation, but also of other cell fate switches.

We employed an abstract model of HIV-1 reactivation in order to utilize analytical tools from dynamical systems theory to explore system behaviour. These tools are increasingly being used in various biological contexts to understand cell fate decisions^10–12^ and such coarse models have proven useful for explanatory purposes in other systems^13,14^. In particular we employ the connectionist model of gene regulation^15^, which has been biologically-verified and widely-employed in multiple different modelling contexts. In order to utilize dynamical systems analysis it was preferable to have a simple continuous regulatory function. Therefore, we used the abstract sigmoid regulatory function that captures the basic behavioural features of the LTR promoter (See Supporting Information section ‘The gene regulatory function’ for full details and justification). The use of such an abstract model is justified since the aim of the model was not to produce accurate quantitative predictions for HIV reactivation but instead to explore fundamental properties that could be extrapolated to other multi-stable systems. If one wishes to generate accurate quantitative predictions for HIV reactivation then a model that is constructed bottom-up from the known components and parameters is more appropriate. One example of such a model is that used by Weinberger and colleagues which is largely based on mass action^9,16^.

## Results

**Figure 2:**
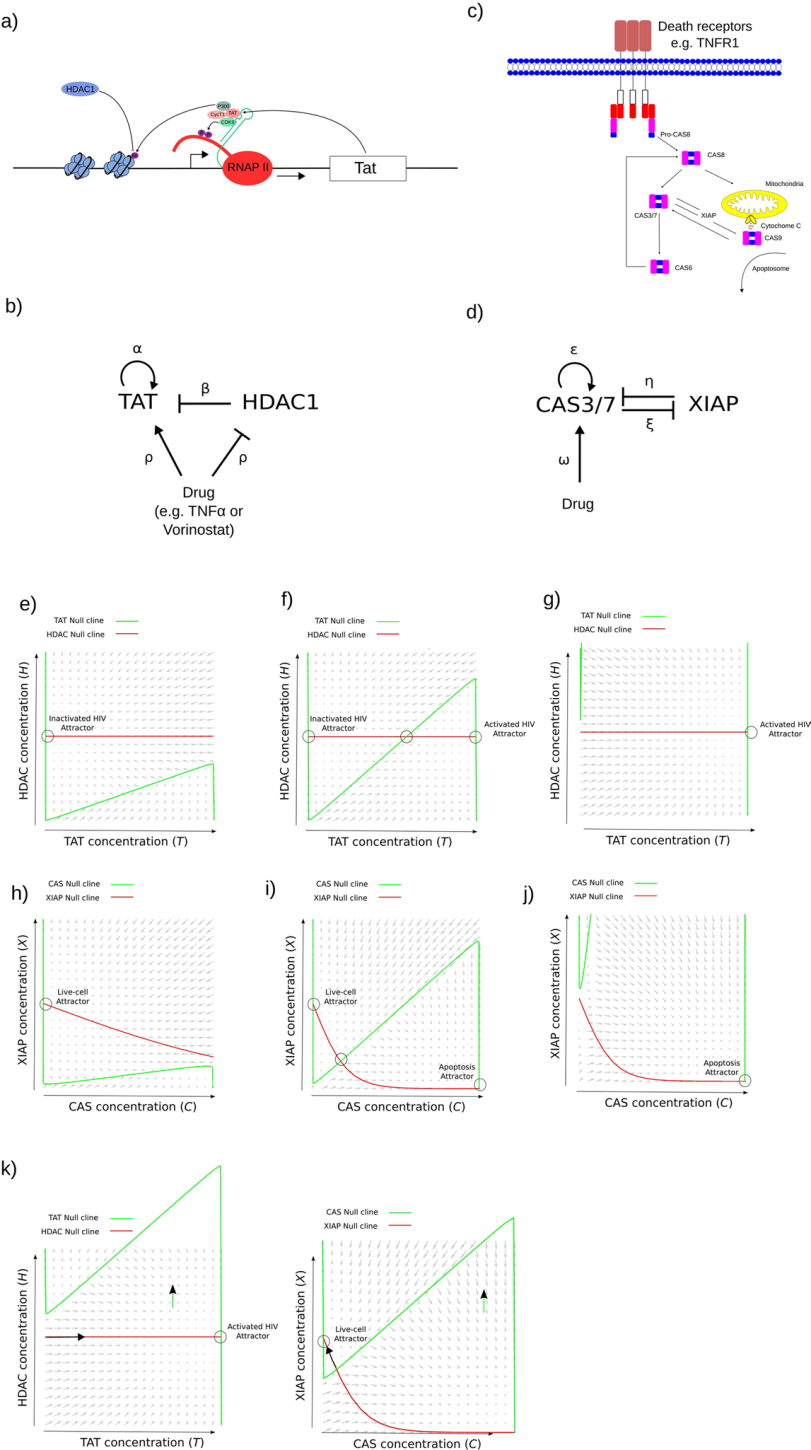
A coarse grained model for HIV reactivation and apoptosis in cancer therapy. (**a**) The TAT positive feedback components in HIV. Phosphorylation (P) and Acetylation (Ac) are shown. (**b**) Regulatory network representing a coarse-grained model of the HIV system with attempted reactivation. (**c**) The core part of the caspase network controlling apoptosis. (**d**) A regulatory network representing a coarse-grained model of the apoptosis system with attempted HIV reactivation. (**e**–**g**) A set of null clines and underlying phase space for one parameter set of the HIV system in the scenario without drug application. The *x*-axis is the concentration of TAT whilst the y-axis represents the level of HDAC. The grey arrows at each position in the phase space describe the behaviour of the system at that particular concentration profile pointing to where the system would evolve to in the next discrete time step. The HDAC null cline is shown in red and the TAT null cline is shown in green. Dashed circles highlight the stable states of the system. (**h**–**j**) An equivalent set of nullclines to e-g but for the apoptosis system. (**k**) An example set of null clines and underlying phase space for the HIV (left) and apoptosis (right) systems in the scenario with drug application. Here the TAT and CAS null clines are translated upwards (green arrow) resulting in the destruction of 2 stable states in the HIV system (left). Cells in the ‘inactive’ stable state now move along the HDAC nullcline towards the ‘active’ stable state (black arrow). The CAS nullcline by contrast is not translated sufficiently to allow for the destruction of the live-cell stable state or unstable node and cells fall back to the live cell attractor (black arrow; right).

To construct the model, we focused on the TAT protein to represent the level of activated HIV-1 since it is under the control of the HIV-1 Long terminal repeat (LTR) promoter (Fig. 2a and b). The TAT protein is crucial for the enhanced efficiency of viral transcription. TAT binds to the transcription activator response (TAR) element located at the 5′ end of the viral RNA transcript and enhances transcriptional activity through Cdk9 induced hyperphosphorylation of RNA polymerase II and through recruiting the p300/CBP and PCAF transcriptional coactivators/acetyltransferases to the promoter resulting in the acetylation of nucleosome 1 and enhanced transcription^17–21^. This behaviour leads to positive feedback of Tat on its own expression which we have therefore included in the model. The host factors YY1 and LSF have been shown to cooperatively recruit histone deacetylase 1 (HDAC1) to the HIV-1 long terminal repeat (LTR) and inhibit transcription^22^. Hence, we modelled the general inhibition of LTR expression and subsequent production of TAT protein by including inhibition of Tat by HDAC1 through the LTR promoter. Tat activation and HDAC1 inhibition inputs integrate into the LTR promoter to define Tat expression in an additive fashion since TAT and HDAC1 promote acetylation or deacetylation at nucleosome 1 at the LTR promoter respectively. The S-shaped curve observed for LTR activation utilizing HDAC1 inhibitors with different IC50s in cell lines demonstrates that cooperativity must be apparent in acetylation control of expression from the LTR^23^. Therefore, we utilized a sigmoidal response function to represent cooperative Tat activation by acetylation of nucleosome 1 at the LTR. Finally, the half-life of TAT from various different HIV-1 subtypes have been measured and all show characteristic exponential decay dynamics^24^. Hence, we have included an exponential decay term for the TAT protein component.

In our model we focus on attempting to reactivate latent HIV using drugs that increase the level of histone acetylation and thus the expression from the LTR. Many different drugs have been used to attempt reactivation that have this effect including TNF-alpha (through NF-kappa-b^25^) and various HDAC1 inhibitors^26^. Hence, we have modeled the application of reactivation drug as a positive input into Tat that represents increasing the relative amount of acetylated nucleosome 1 (Results are confirmed implementing the drug as an inhibitor of HDAC1 - see later). One of the major side effects that results from HIV-1 reactivation drugs is cytotoxicity. TNF-alpha for example is known to activate the caspase cascade through the TRADD domains of the TNF receptor, whereas HDAC inhibitors such as Vorinostat (SAHA) and MS-275 have been shown to cause death of normal mesenchymal stem cells through the activation of Caspase-9^27,28^. Therefore we focused on apoptosis to represent side effects caused by drugs used to attempt HIV reactivation.

Due to the need for apoptosis to be tightly regulated, multiple feedback loops and control points are employed in the caspase cascade. Caspases are produced as inactive pro-caspases which require proteolytic activation. A representation of the caspase network is shown in Fig. 2c. Multiple positive feedback loops have been identified in the caspase cascade which allow it to act in an all-or-nothing bistable manner. The effector Caspase-3 is known to indirectly lead to activation of Caspase-8^29,30^. The most well characterized mammalian inhibitor of apoptosis (IAP) is XIAP which can inhibit Caspase-3 and -7 by binding to their active catalytic sites with low nanomolar efficiency^31–33^. Caspase-9 is known to directly cleave and activate Caspase-3 and -7 as part of the apoptosome^34^. A positive feedback loop also exists between Caspase-3 and -9 since Caspase-3 inhibits XIAP which in turn inhibits Caspase-9^34,35^.

We focused our abstracted network on the effector caspase-3 and 7 since they lie at the crux of the caspase activation cascade integrating the multiple feedback loops (Fig. 2d). The level of caspase 3/7 in this model therefore represents the apoptotic state of the cell. We captured the positive feedback behaviour of the caspases through including both a positive feedback loop on caspase 3/7 (representing the caspase-8 positive feedback loop) and through an inhibition of caspase-3 onto XIAP (representing the caspase-9 positive feedback loop). To date XIAP is the only known inhibitor of these caspases that can do so at physiological concentrations and hence we modelled the inhibition of caspase-3/7 with a XIAP component^36^. The effector caspases-3 and -7 half-lives have been measured to be 8 and 11 hours respectively and the dynamics of inflammatory caspase-1 demonstrate exponential decay^37^. Hence, we have employed a decay term for the caspases that represents such exponential decay.

Activation of the pro-caspases involves dimerization and often oligomerisation, followed by cleavage into a small subunit and large subunit^38^. This cooperative dimer/oligomerisation event means that a sigmoid type response function is suitable for modeling caspase activation and hence we employed this for the caspases. This cooperatively partly explains the well-known bistable (ultrasensitivity) behaviour^39^ of the caspases. Active caspase production is limited by the quantity of pro-caspase present and thus we have again assumed a saturating sigmoid function. An additive model of regulation is appropriate in this scenario since XIAP can be thought to directly titrate the number of active caspase 3/7 molecules through binding the active catalytic site. Cooperativity is apparent due to the requirement for oligomerization in the caspase-3,-7,-8, and -9 activation^38^ and therefore a sigmoidal shape activation curve is appropriate. Saturation is also apparent due to various intrinsic limitations including the amount of inactive procaspase-3/7 available in the cell.

In order to explore the behaviour of such a system we employed tools derived from dynamical systems theory. Specifically, we utilized phase spaces to determine whether attractors (stable states) exist in the system and if so what is the shape of their corresponding basins of attraction. A nullcline of a variable defines where (at what states of the system) its rate of change is zero. Fixed points of a system can be determined where nullclines intersect since at these points none of the concentrations of the components of the system are changing. We generated a set of nullclines for a representative set of parameters for each state of the system (no drug and drug-applied). The nullclines derived from the model equation sets (See methods and Supporting Information) were thus overlaid on top of phase spaces resulting from dynamical simulation of the system at every possible state (Fig. 2e–k; see Supporting Information).

The nullclines analysis demonstrates that there exist 3 possible configurations of the null clines for both the HIV and apoptosis systems depending on the parameters and application of drug (Fig. 2e–j). We performed stability analysis to confirm that these are all possible configurations and to determine whether these fixed points are stable attractors or unstable nodes (See Supporting Information section ‘Stability analysis’ and Figure S4). Two of these fixed points correspond to stable attractors for the HIV-inactivated and HIV-activated scenarios (dashed circles; or live-cell and apoptosis scenarios in the apoptosis system). A third fixed point corresponds to the unstable saddle node at the apex of the null clines defining the boundaries of the basins of attraction (non-dashed circle). Two basins of attraction drain to either the HIV-inactivated or HIV-activated attractors (or live-cell and apoptosis attractors).

Considering the non-drug applied scenario, the only relevant configurations for the HIV system are shown in e and f (and equivalently h and i for the apoptosis system) since in the configuration shown in g (j for apoptosis) HIV will always reactivate and thus the application of drug is irrelevant. If we activate the TAT component by a drug, as demonstrated in Fig. 2k (left), the unactivated basin of attraction is destroyed (along with the attractor and unstable saddle node) and any cell with dormant HIV now follows the HDAC nullcline towards the HIV-activated attractor (as illustrated by the black arrow in Fig. 2k; also see underlying phase space flow). However, TAT activation by the drug is likely to be only temporary due to drug decay. Hence, at some point the TAT null cline declines back to its original position like in Fig. 2e and f. Depending on how far cells have migrated along the HDAC nullcline they may have accessed the HIV-activated basin of attraction even though drug activity has switched off. Alternatively, if they have not passed the point of the unstable saddle node then they are still in the HIV-inactivated basin of attraction and will fall back to that attractor.

A simple suggestion for an alternative strategy for increasing the frequency of HIV reactivation arises from this analysis. The phase spaces suggest that continuous delivery of low concentrations of drug rather than discontinuous delivery of higher concentrations (with equivalent drug exposure) may result in a much higher frequency of reactivation. The longer the cells have to pass along the HDAC nullcline trajectory the more chance they have of accessing the HIV-activated basin of attraction. One way to achieve this would be to maintain the ‘channel’ open for passage to the HIV-activated attractor by not switching off the activation, so that the TAT nullcline will stay in the configuration shown in Fig. 2k (left). The important point here is that while the TAT nullcline is moved sufficiently by the low levels of drug to maintain a configuration such as that in Fig. 2k (left), the CAS nullcline maintains a configuration like that in Fig. 2k (right) since it is less strongly activated by the drug so that cells do not undergo apoptosis. Dose response curves such as those in Fig. 1a suggest that this scenario is possible since desired biological response occurs at lower concentrations than detrimental biological response.

**Figure 3:**
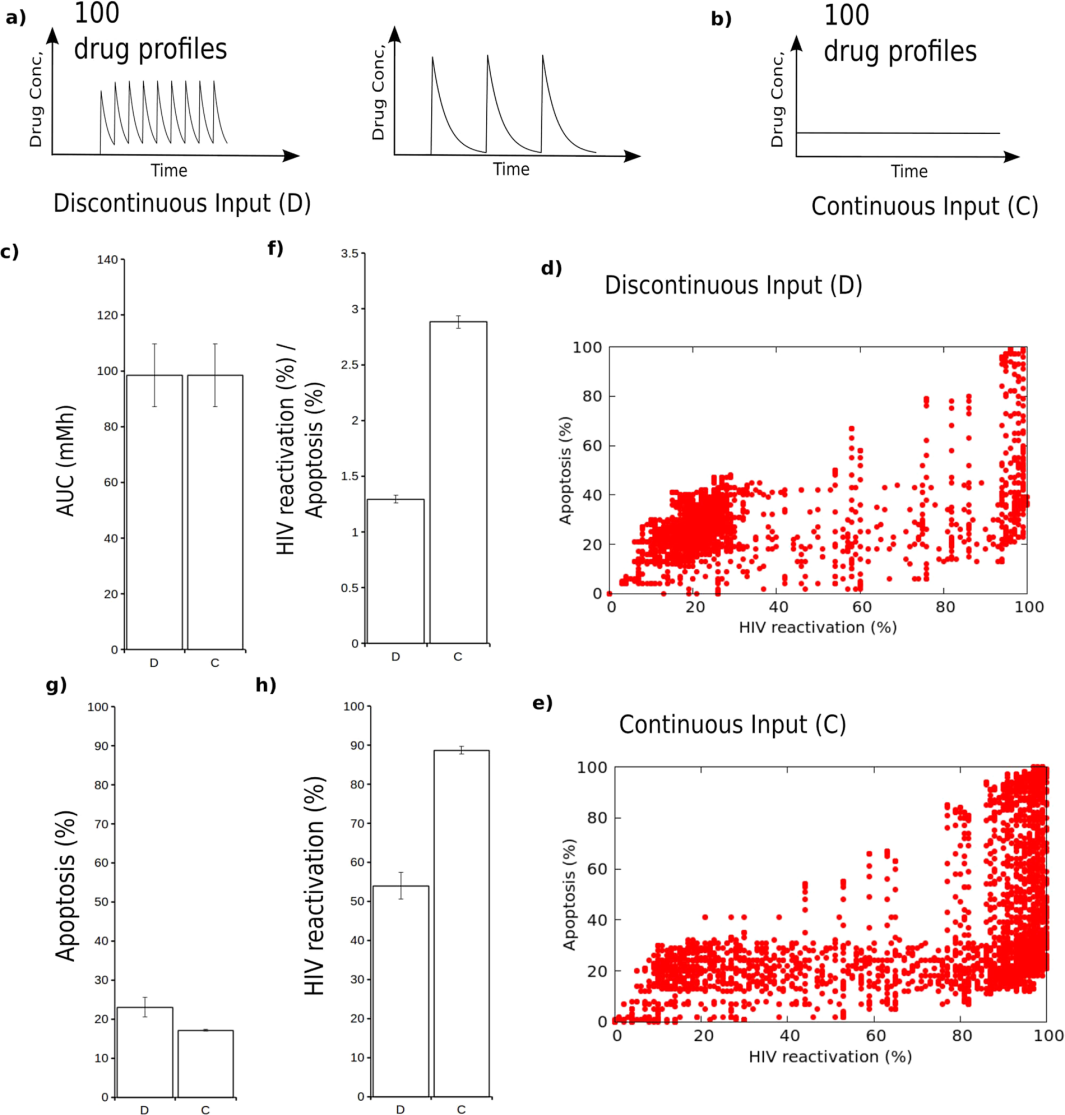
Continuous drug delivery offers superior effectiveness/side-effect balance in cellular state switching. (**a**) examples of 2 different dose scheduling profiles for the discontinuous case. Multiple profiles were tested in each regimen. (**b**) For each discontinuous dose scheduling profile, we calculated the corresponding continuous dosing schedule with the same area under the curve (AUC). (**c**) Measuring the total AUC for the discontinuous and continuous regimens demonstrates that they are identical. AUC is measured in milliMolar Hours. The Standard error is plotted. (**d** and **e**) A scatter plot for HIV reactivation (*x*-axis) and Normal cell apoptosis (*y*-axis) in the discontinuous (**d**) or continuous (**e**) regimen. Each red dot is one particular drug at one particular dose in the dosage range (See Supporting Information). (**f**) Quantification of HIV reactivation relative to apoptosis for all points in e and f where apoptosis is not equal to 0. Standard error is shown. p < 0.0001 that there is a difference between the two groups using a two-tailed unpaired *t-*test. (**g**) Quantity of apoptosis for the two regimens when we identify the best (maximal HIV reactivation with apoptosis below 20%) dose for each drug. The average apoptosis for each drug is plotted on the *y*-axis. The Standard error is plotted. (**i**) Quantity of HIV reactivation for the two regimens when we identify the best (maximal HIV reactivation with apoptosis below 20%) dose for each drug. The average HIV reactivation for each drug is plotted on the *y-*axis (p < 0.0001).

To test this hypothesis, we randomly selected 100 hypothetical drugs that more strongly activate the HIV system than the apoptosis system (i.e. ρ ≫ ω - see Methods). Drugs with this property are likely to be the ones that will conform to the phase space constrictions described. The drug half-lives were also randomly selected (See Methods). Once we had screened for a set of hypothetical drugs with the appropriate properties, we then tested them in different dosing regimens. For each drug we chose dosing profiles for a discontinuous case where the number of bouts of application and the length of time between bouts were selected randomly (2 examples are given in Fig. 3a; See Methods and Supporting Information). For each discontinuous dosing regimen, we then constructed an equivalent continuous dosing regimen (example is given in Fig. 3b). Importantly the continuous dosing regimen was selected so as to have the exact same drug exposure (area under the curve - AUC) as its equivalent discontinuous counterpart (Fig. 3c). We then applied these drugs with their specific dosing regimens at increasing doses (see Methods; at all times with an equal AUC between discontinuous and continuous cases) to groups of 100 HIV dormant cells (Each cell has a different gene regulatory network parameter set - see Methods and Supporting Information). We plotted the proportion of HIV dormant cells reactivating HIV or undergoing apoptosis for each drug at each dose of the dosage range. The results of the discontinuous case are shown in Fig. 3d and demonstrate that there is a general correlation between HIV reactivation and apoptosis. The results of the continuous case are shown in Fig. 3e and show a similar distribution to that of the discontinuous case. However, quantifying the average results of all points from each scatter plot and measuring HIV reactivation relative to apoptosis reveals a significant better desired-effect/side-effect trade off for the continuous case compared to the discontinuous case (Fig. 3f).

To explore this effect further, for each drug and dosing regimen we recorded only the dose that gave the best response (highest percentage of cells activating HIV with apoptosis below 20%) for the continuous and discontinuous regimens in 100 HIV dormant cells. We then averaged the amount of HIV reactivation and apoptosis for each of the regimens. Unsurprisingly the amount of apoptosis is similar for both continuous and discontinuous cases as shown in Fig. 3g which occurs at about the 20% level where we constrained successful solutions. However, when we look at the frequency of HIV reactivation we find that it is significantly higher (approximately 50%) in the continuous case relative to the discontinuous case (Fig. 3h). This suggests that for most drugs used in disease treatment scenarios it will be possible to find a continuous dosing scenario that outperforms or at least performs as well as a discontinuous scenario with the same drug exposure (concentration-time AUC).

**Figure 4:**
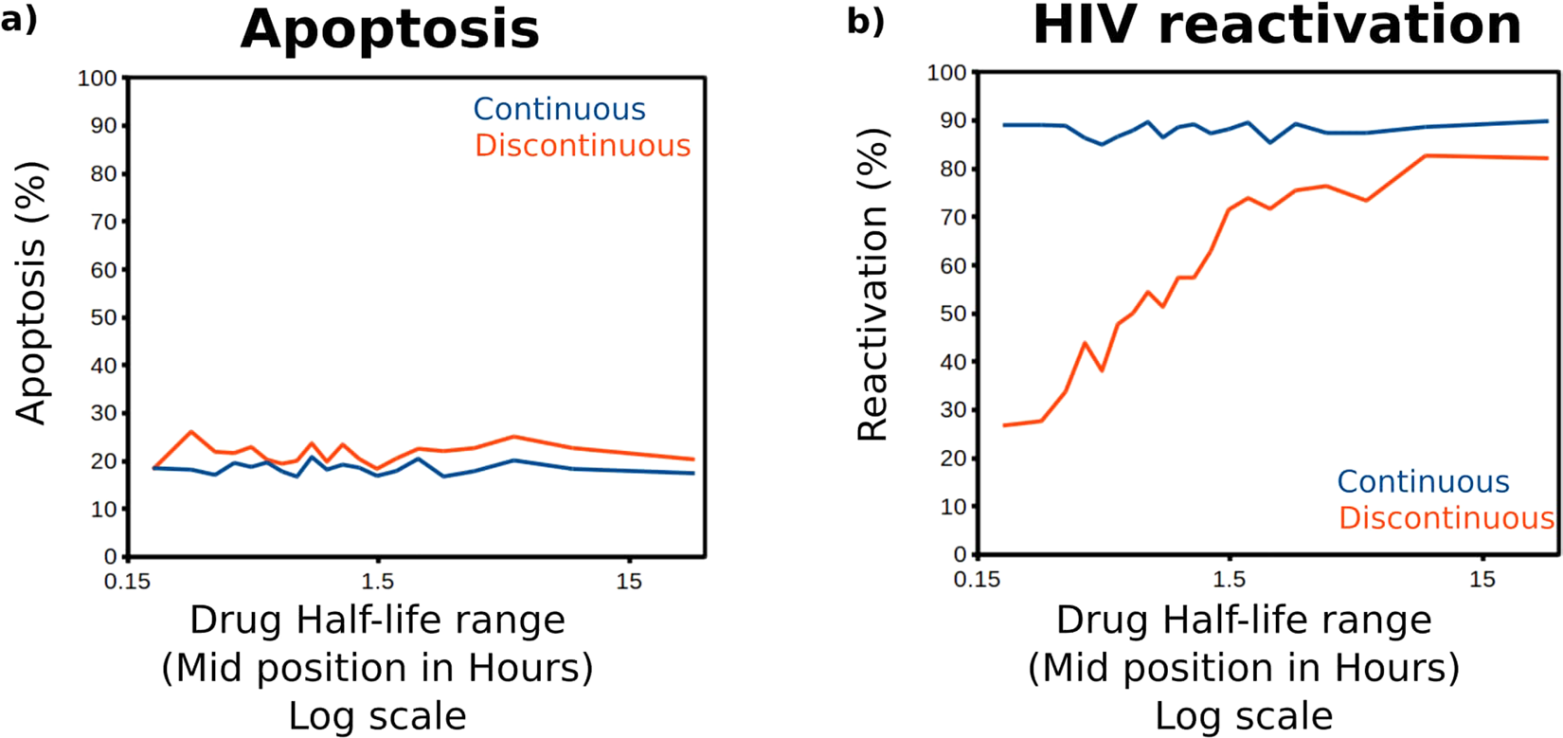
The superiority of the continuous drug delivery strategy is limited to drugs with short half-lives. HIV reactivation and apoptosis frequency after screening for drugs with half lives in different ranges (See Supporting Information). (**a**) Apoptosis frequency (*y*-axis) in different half life ranges (*x*-axis defines the midpoint of the sampled range). The continuous regimen is shown by the blue line and discontinuous regimen by the red line. (**b**) HIV reactivation frequency (*y*-axis) in different half life ranges (*x*-axis defines the midpoint of the sampled range). The continuous regimen is shown by the blue line and discontinuous regimen by the red line.

We explored how this result is affected by drug half-life which would be expected to affect how long the nullcline configurations permissive to switching are maintained. We performed the same analysis as in Fig. 3g and h but randomly selecting for drugs using different half life ranges (See Supporting Information). The results for apoptosis shown in Fig. 4a show that the drug half-life has little effect. However, the results for HIV reactivation (Fig. 4b) show that the improved performance of continuous drug application compared to discontinuous application (with the same drug exposure) occurs to a greater extent at shorter drug half lives. As drug half life increases the effect is gradually lost with half lives of greater than several hours giving similar results between the two regimen types.

Often it is not pragmatic to continuously deliver a drug. A more realistic scenario then which reflects the choices clinicians/scientists have in the clinic/laboratory is changing the frequency of dosing. Hence, we explored the model further by analyzing the effects of our 100 hypothetical drugs when added to the the cells over a range of frequencies with drug exposure kept the same between each test. The results shown in Figure S5 (See Supporting Information section ‘Comparing the desired-effect/side-effect balance with different frequencies of interval dosages’) show that the more frequently that drugs are applied, the better the trade-off between desired-effect and side-effect.

Extreme stochastic noise is expected at the LTR due to the typically single copy number of integrated HIV. To explore the effect of stochastic noise on the results presented here we have adapted the model to mimic the stochastic noise that occurs at the LTR promoter (See Supporting Information section ‘Exploring the effects of stochastic noise on the model’). The results shown in Figures S7 and S8 confirm that they are robust to the addition of a basic implementation of stochastic noise to the LTR promoter. Analytically this result can be understood when exploring the effect of stochastic noise on the phase space and nullclines (Figure S9).

In order to validate the concept that more continuous drug delivery can result in superior efficacy even with the same drug exposure, we focused on HDAC inhibitors that have been extensively examined in recent times as potential HIV reactivation agents. The mechanism of action of HDAC inhibitors is clearly represented by the model presented here since inhibiting HDAC should have a similar effect to activating TAT by hypothetical drug (i.e an indirect inhibitor of an inhibitor versus direct activator). Nonetheless we confirmed that the same qualitative results were observed when implementing the hypothetical drug in our screen as an inhibitor of HDAC rather than an activator of TAT (See Supporting Information section ‘Implementing the drug as an inhibitor of HDAC instead of an activator of TAT’ and Figure S10).

**Figure 5:**
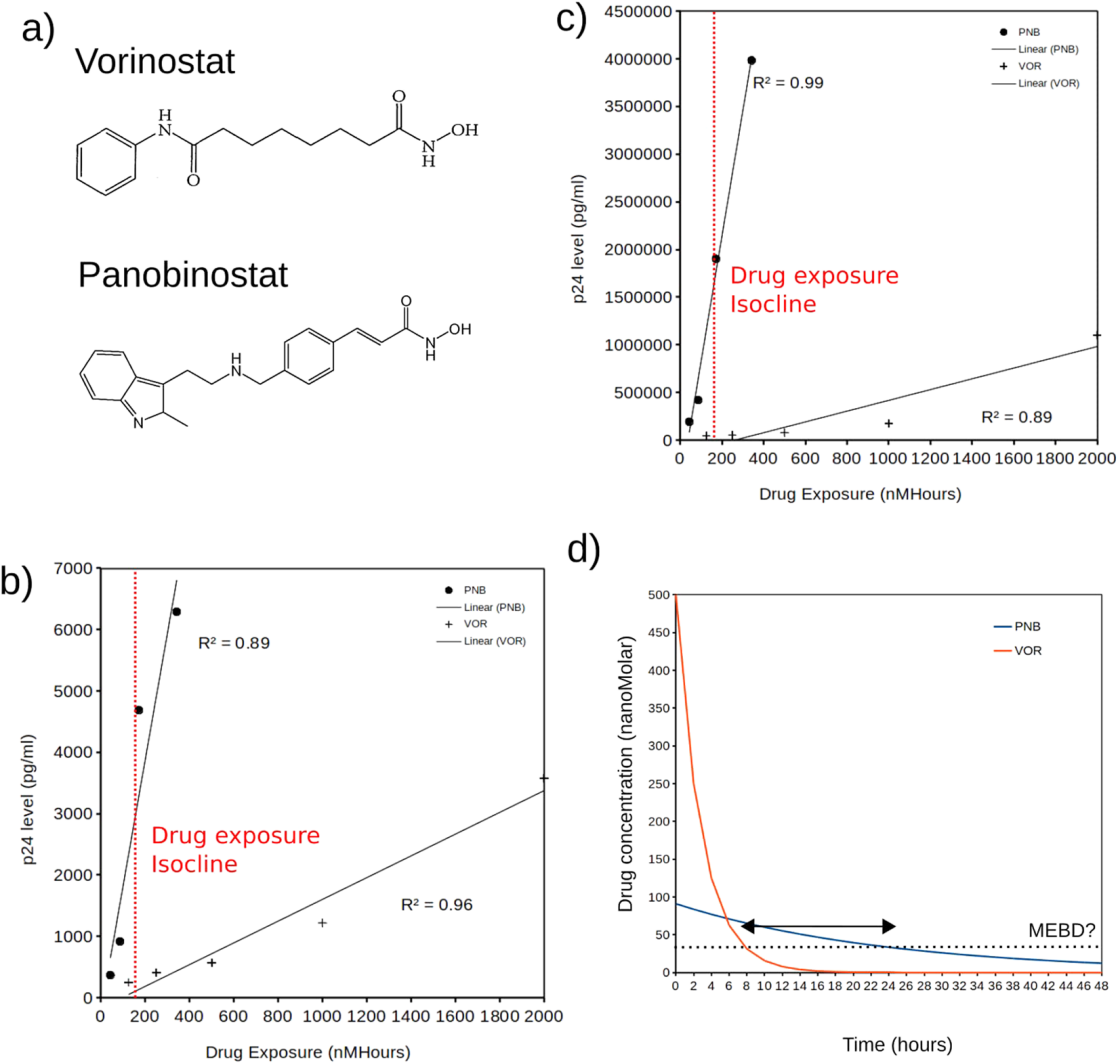
Validation of the concept through exploring the effects of the Histone Deacetylation drugs, Vorinostat (VOR) and Panobinostat (PNB) in cell culture (Original data from Rasmussen *et al.*, 2013). (**a**) Chemical structures of VOR and PNB (**b**) p24 assay after treatment with VOR and PNB in latently infected U1 cells. Drug exposure was reverse engineered using the drug half-lives (2 hours for VOR and 16 hours for PNB). A linear trend-line was fit to the data. A drug exposure isocline is shown. (**c**) p24 assay after treatment with VOR and PNB in latently infected ACH2 cells. Drug exposure was reverse engineered using the drug half-lives (2 hours for VOR and 16 hours for PNB). A linear trend-line was fit to the data. A drug exposure isocline is shown. (**d**) Demonstrating drug profiles for VOR and PNB with identical exposure (AUC is approximately 1000 nM Hours) assuming starting doses of 91 nM for PNB and 500 nM for VOR. The long tail of the PNB profile could potentially maintain the channel open between inactive and activated HIV attractors (i.e. above MEBD as indicated by the dashed line - see later). The double headed arrow indicates the region where PNB would be maintaining the channel open for HIV switching whilst it would be closed with VOR.

We compared 2 of the most extensively explored HDAC inhibitors Panobinostat and Vorinostat because they are in the same mechanism-of-action class, both being hydroxamates with an active site occluding cap and a zinc ion chelating region separated by a linker resulting in similar nanomolar IC50s against a broad range of HDACs^40^ (Fig. 5a). However, they have markedly different half-lives with Vorinostat being measured at 2 hours^41^ whilst Panobinostat being measured at approximately 16 hours^42^. As we have shown in Fig. 4, we would expect these differences in half-lives to have a significant effect on their abilities to reactivate HIV. Indeed, a recent study exploring a panel of HDAC inhibitors for HIV activation using an *in vitro* latency assay demonstrated that Panobinostat displayed a significantly superior potency compared to Vorinostat^43^. We reverse engineered the drug exposures used in this study (over the 48 hours used in the assay) utilizing the known half-lives of the drugs and displayed the results as a scatter plot of drug exposure versus potency (Fig. 5b,c). Firstly, the results show that there is a very strong linear correlation between drug exposure and effect (excluding highest saturating dose of PNB). Secondly the results clearly show that Panobinostat is superior to Vorinostat even when drug exposure is the same. We suggest that Panobinostat offers superior potency to Vorinostat due to its longer half-life and thus more continual exposure in this assay allowing cells to pass along the HDAC null cline into the activated HIV basin of attraction before the drug drops below the concentration required to maintain the channel open (Fig. 5d; above Minimum effective bifurcating dose - see later). By contrast we suggest that Vorinostat pushes cells along the HDAC nullcline but that those cells decay back to the inactivated HIV attractor before they can cross into the activated HIV basin of attraction due to the short half-life of the drug. This may also offer an alternative explanation as to why in various studies using vorinostat, production of free virus from latently infected cells was rarely observed, even when an increase in CA-US HIV RNA was clearly detected^44–47^. If CA-US HIV RNA has a long enough half-life, then it will be detected at significantly increased levels as a relic of the promoter activation even though the virus was never reactivated.

## Discussion

In summary we have demonstrated that continuous drug delivery at low doses is a potentially effective strategy for disease treatment. The same drug exposure that is effective in switching on latent HIV with minimal side effects using continual dosing would not necessarily be effective using intermittent dosing. The difference in the effect of continuous versus discontinuous dosing at identical drug exposure can be understood when one considers how the configuration of the phase spaces and nullclines change over time in combination with the effects of saturating activation. In the intermittent dosing scenario, there is a state of flux between the phase space/nullcline configurations shown in Fig. 2k left (drug recently applied) and Fig. 2f (low drug concentration due to decay). TAT production saturation results in a maximum rate that a cell can move along the HDAC nullcline. Therefore, the fact that the intermittent dosing scenario spends time in the phase space/nullcline configuration shown in Fig. 2f during times of low drug concentration is not compensated by more cells entering the activated HIV basin of attraction during times of higher drug concentration. By contrast a continuous dosing scenario with identical drug exposure will be fixed in the phase space/nullcline configuration shown in Fig. 2k such that the ‘channel’ for state switching (cells move along the HDAC nullcline from inactivated HIV attractor to activated attractor) will remain open, resulting in a higher frequency of HIV reactivation.

**Figure 6:**
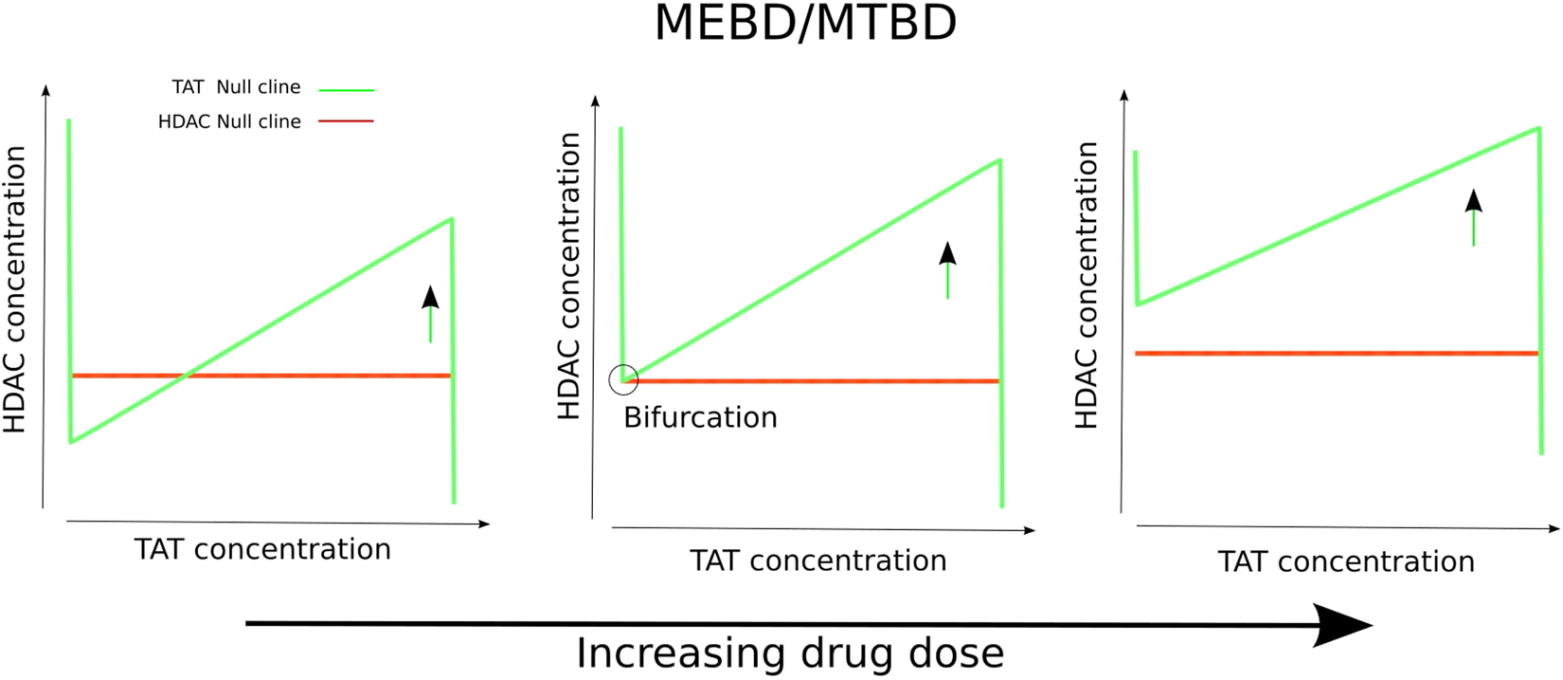
The definitions of Minimal effective bifurcating dose (MEBD) and Maximum tolerable bifurcating dose (MTBD). (from left to right) Increasing the dose of the drug translates the TAT null cline upwards. (center) Minimum effective bifurcating dose occurs at a dose of drug that causes the apex (dashed circle) of the TAT null cline to cross the HDAC null cline. MTBD involves the same configuration but with the TAT null cline replaced with the Cas nullcline and the HDAC nullcline replaced with the XIAP null cline. MTBD occurs at a dose where the Cas Nullcline crosses the XIAP nullcline.

Importantly this effective low continual dose likely occurs much further below the drug dose classically defined as the ‘Minimum effective dose’. The minimum effective dose is typically defined as the minimum dose of a drug that will produce the desired/undesired outcome in some fraction of the subjects to which it was applied and is often estimated without taking into account the number of bouts of drug that have been applied or the time interval(s) between them^48^. We suggest that this concept (and by similar analogy MTD) is largely ambiguous since a whole range of doses applied at different frequencies can potentially fit this definition. We suggest alternative non-ambiguous definitions of the MED and MTD which we term ‘Minimal Effective Bifurcating Dose’ (MEBD) or ‘Minimal Tolerable Bifurcating Dose’ (MTBD) respectively which are the doses that result in a bifurcation in the phase space allowing the biological responses to occur (Fig. 6). In our case this would be the dose when the TAT null cline (for MED) or CAS nullcline (MTD) had been translated sufficiently to leave only the ‘TAT/CAS on’ stable point intact. MEBD is likely to occur at doses that have classically been defined as sub-therapeutic (i.e. at a much lower concentration than MED), since MED is rarely measured using a continuously applied drug. We therefore speculate that in many therapeutic interventional settings there may exist a much extended scope for lowering drug dose (and applying continuously) than previously thought yet increasing effectiveness and reducing side effects simultaneously.

The principal shortcoming of the model presented here is its abstract nature which means that it is difficult to make precise quantitative predictions for HIV reactivation. At the same time however, the abstract nature of the model offers the advantage that the basic findings can be extrapolated to other systems. In particular the general message here applies to any system where both the desired transition and side effects are defined by attractors in phase space which likely includes most systems with multiple cellular states. Therefore, in any drug application or experimental setting where the aim is to generate a cell type switch the described strategy may be of use. Implementations of the described strategy in disease treatment scenarios include intravenous rather than oral drug delivery, focusing on improvements in pharmacokinetics, drug half-life and methods to aid slow drug release such as pegylation and liposomal delivery^49^.

Finally, for the HIV reactivation setting, the specific useful conclusion that results from this work is the suggestion that focusing on the longer half-life Panobinostat is likely to prove more fruitful for reactivation of HIV than the shorter half-life Vorinostat even at lower doses that may at first sight appear subtherapeutic. Determining MEBD for Panobinostat could be achieved through *in vitro* studies using a close proxy for HIV reactivation at cellular resolution. The drug exposure would need to be determined precisely through the Area under the curve (AUC) measurements. The MEBD could then be used in combination with other pharmacokinetic parameters to inform on an appropriate dosing schedule in a clinical setting that should maximize latent HIV reactivation whilst minimizing side effects.

## Materials and Methods

### Model construction

Full details of the model including the biological meaning of the parameters, sampled parameter ranges, assumptions behind the model, units, timescales and nullcline equations are given in Supporting Information. We utilized a sigmoid function to describe the input-output relationship of the components of the model. We confirmed that the qualitative results were not dependent on the exact regulatory function used by also using a Hill function (See Supporting Information section ‘Exploring the model with an alternative hill function’ and Figure S11).

The basic equations of the model are as follows:

1 $$\frac{dT}{dt}=\frac{{K}_{M}}{(1+{e}^{(\varphi -\chi (\rho +\alpha T-\beta H))})}-{\gamma }_{T}T,$$

2 $$\frac{dC}{dt}=\frac{{K}_{M}}{(1+{e}^{(\varphi -\chi (\omega +\varepsilon C-\eta X))})}-{\gamma }_{C}C,$$

3 $$\frac{dH}{dt}=\frac{{K}_{M}}{2}-{\gamma }_{H}H,$$

4 $$\frac{dX}{dt}=\frac{{K}_{M}}{(1+{e}^{(\varphi -\chi (-\xi C))})}-{\gamma }_{X}X,$$

where *T, C, H* and *X* are the concentrations of TAT, CAS, HDAC and XIAP respectively. *α, β, ε*, *ρ, η* and *ω* are regulatory parameters, *χ* is the input scalar, *φ* in the universal scalar which control the shape of the regulatory function and, *γ*_*T*_, *γ*_*C*_, *γ*_*H*_ and *γ*_*X*_ are the gene product decay parameters. *ρ* and *ω* are set to 0 when there is no drug application. *K*_*M*_ represents the maximum production rate of a particular component which is set to 1 throughout this work. The origin of the parameters and their units are described in greater detail in Supporting Information. When we perform numerical simulations of the model with discrete time we use difference equations that correspond to equations 1–4.

### Hypothetical drug selection

We randomly selected 100 hypothetical drugs for testing in the continuous and discontinuous dosing regimens. The half-life of the hypothetical drug (*κ*) and the strength by which the hypothetical drug activates both the TAT (*ρ*) and the CAS (*ω*) are considered properties of the hypothetical drug. These parameter values were selected at random for each hypothetical drug (See Supporting Information for exact ranges). Importantly our random parameter selection was biased so that *ρ* ≫ *ω* in order to specifically select for drugs that activate HIV reactivation more than apoptosis (and therefore have some degree of therapeutic window). Note that identical sets of drug schedule profiles and half-lives are used for both the TAT and CAS systems with the only change coming from the strength of drug activation parameter (*ρ* or *ω*).

### Random drug schedule profiles

Once we had selected suitable hypothetical drug parameter sets, we then tested these hypothetical drugs with different drug schedule profiles in the discontinuous and continuous delivery regimens. For the discontinuous delivery for each hypothetical drug we randomly selected a drug schedule based on the number of drug bouts (drug applications) and time between bouts. The number of drug bouts was randomly selected uniformly between 1 and 10 and the time between bouts between 1 and 200 hours. The dose at any one particular time point is given by the equation

5 $${D}_{t}=\sum _{1}^{N}{D}_{0}{\mu }^{(t-L(n-1))},$$

where *t* is the current time point, *L* is the time per bout, *N* is the total number of bouts up until time point *t*, *n* is the current bout being processed (between 1 and *N* of the function), *D*_0_ is the initial dose applied and *μ* is the drug decay parameter. We measured the drug exposure for the discontinuous schedule by calculating the area under the curve (AUC, in millimolar hours) and used that to define the dose of the continuous schedule. Specifically, the dose of the continuous schedule was defined as

6 $${D}_{t}=\frac{E}{NL}\,,$$

where *E* is the drug exposure for the discontinuous regimen, *N* is the number of bouts, *L* is the time per bout in the discontinuous regimen and *D*_*t*_ is the dose at the current time point (does not change throughout simulation). The continuous dose does not change throughout the simulation. This calculation results in an identical drug exposure (AUC) for both the discontinuous and continuous drug delivery regimens as demonstrated in Fig. 3c.

### *In silico* assay for HIV reactivation efficiency and apoptosis

The *in silico* assay that we employed mimics a cell culture model of a disease though could also represent testing a drug in a population in individuals such as in a clinical trial (See Supporting Information). For each hypothetical drug and its specific regimen (discontinuous and continuous) and scheduling profile (N and T) we applied escalating (doubling at each test) doses of drug from 0.1 nM to 0.1 M. For each test (specific regimen, schedule and dose) we then simulated 100 HIV dormant cells over NT hours and examined whether the TAT or CAS components had reached 80% of their maximum concentration (max concentration is calculated as 1/*γ* Molar). If the TAT or CAS components had reached 80% of their maximum concentration then we considered that the cell had reactivated HIV or undergone apoptosis respectively. The proportion of HIV dormant cells that reactivated HIV or underwent apoptosis was calculated and this data was plotted as scatter plots in Fig. 3d and e.

To find the best performing dose for each drug in each of the different regimens for each of the tests at different doses above we asked which gave the highest HIV reactivation whilst maintaining apoptosis below 20%. We then recorded the % HIV reactivation and % apoptosis in the best performing dose. The resulting data per drug was averaged for each regimen and used to make Fig. 3g and h.

### Half-life range exploration

In order to explore the effect of hypothetical drug half-life on the better disease treatment results of continuous regimens over discontinuous regimens at the same AUC we performed the hypothetical drug screen but limiting the randomly generated drugs to different half-life ranges. Specifically, we explored drugs with decay parameters (μ) in 0.05 t^−1^ block windows from 0 t^−1^ to 1 t^−1^ which maps to half-lives (κ) from 19 minutes to 27 hours (See Supporting Information for the relationship between decay parameter and half-life). i.e. we generated hypothetical drugs with decay parameters between 0 t^−1^ and 0.05 t^−1^ then between 0.05 t^−1^ and 0.1 t^−1^ etc. For each ensemble of selected hypothetical drugs, we performed the best performing dose analysis and averaged the % HIV reactivation and % apoptosis for each regimen as above. The midpoint of the tested half-life range is plotted in Fig. 4.

### Reverse engineering Vorinostat and Panobinostat drug exposure

We used the published values for the half-lives of Vorinostat (2 hours^41^) and Panobinostat (16 hours^42^) to reverse engineer the drug exposure in each scenario tested. In the described p24 assay drugs were applied once and the assay performed 48 hours later^43^. Therefore, drug exposure can be calculated by

7 $$E={0.5}^{(t/{\kappa }_{D})}{D}_{0},$$

where *E* is the total drug exposure, *D*_0_ is the concentration of the drug at time point 0, t is the current time point (we increment in hours) and *κ*_*D*_ is the half-life of the drug in question. We then plotted drug exposure against the published p24 responses.

## Electronic supplementary material

Supplementary Information
