# Author Correction: A strategy for effective latent HIV reactivation using subtherapeutic drug doses

**DOI:** 10.1038/s41598-018-21977-8

**Published:** 2018-03-06

**Authors:** James Cotterell, G. Gregory Neely

**Affiliations:** 1 The Garvan Institute for Medical Research, 384 Victoria Street, Darlinghurst, Sydney, NSW 2010 Australia; 2 The Dr. John and Anne Chong Lab for Functional Genomics, Charles Perkins Centre and School of Life & Environmental Sciences, The University of Sydney, Camperdown, NSW 2006 Australia

Correction to: *Scientific Reports* 10.1038/s41598-017-00097-9, published online 30 November 2017

The original version of this Article contained an error in the spelling of the author G. Gregory Neely, which was incorrectly given as Greg Neely. This has now been corrected in the PDF and HTML versions of the Article.

