# Supplementary material for: A strategy for effective latent HIV reactivation using subtherapeutic drug doses
Source: Sci Rep. 2017 Nov 30;7:16644. doi: 10.1038/s41598-017-00097-9 (PMC5709488; doi:10.1038/s41598-017-00097-9)
Supplement: Supplementary file 1 — Supplementary Information [file 41598_2017_97_MOESM1_ESM.docx]

A strategy for effective latent HIV reactivation using subtherapeutic drug doses.

Cotterell and Neely

**Supporting information**

**Model Context and Pharmacokinetic metrics**

Our model can be applied to cell state changes in general which is useful since the ability to control cell state changes has utility in many different contexts. For example this may be in order to control the transdifferentiation of stem cells in culture or to alter the disease state of a patient. When aiming to control patient disease state the model has applicability for guiding scheduling in the clinic and the effector is specifically a drug. When considering the context of altering patient disease state, the most commonly used pharmacokinetic metrics can be treated as follows.

Dose, dosing interval, C_max_, t_max_, C_min_, concentration and area under the curve (AUC) can all be treated in the same way as would be done for standard pharmacokinetic setup. The only difference is that in the current setup AUC is not normalized to the volume of distribution since in this work we assume that all contexts have the same volume of distribution (5L in an average human). In the simplest model where bioavailability is not taken into account (like it is not here), it can be assumed as 100% as would be the case with intravenous delivery of a drug or cells exposed to an effector in culture. Elimination half-life and elimination rate constant are interdependent and thus only the elimination half-life parameter needs to be utilized which is implemented in our model as the drug degradation rate. For the continuous drug delivery case we can assume that infusion perfectly balances elimination to maintain a continuous blood concentration. For the discontinuous interval dosing case we can assume an infusion rate of 0 such that blood drug concentration is entirely controlled by the initial dose and elimination rate. Because we are considering a uniform volume of distribution for the drug, clearance always has a perfect correlation with elimination and thus can be fully described by the drug half-life. In the simplest model, fluctuation can be excluded though extensions of the model to take these features into account can be included as necessary.

**The Gene regulatory function**

Due to the abstract intention of the model, it was not essential to use a specific type of regulatory input-output function. Nevertheless it was still desirable that the regulatory function represented the real world situation as closely as possible. Therefore we used a pragmatic continuous sigmoid function that could be used in combination with tools from dynamical systems theory yet still capture the core features observed for the regulatory control of the components in our system. These continuous type functions are well-known to have advantages for analytical studies compared to the more commonly used Hill-type functions that suffer from numerous problems. For instance when a Hill coefficient of 1 is used in the Hill type function (Giving the Michaelis-Menten function) both infinite values and negative values are produced as demonstrated in figure S1. Where Hill coefficients >1 are used, other problems arise such as strong activation of components from an inhibitory input. The effect of a discontinuity on dynamical systems analysis can be seen when we plot the phase space and null clines for the Michaelis-Menten function (figure S2 left) where erratic behaviour in the phase space is observed (This phase space is the Michaelis-Menten equivalent to the sigmoidal phase space shown in figure 2k). As can be seen the problem can be corrected with the use of a heaviside function that prevents negative outputs from the regulatory function (figure S2 right and see section ‘**Exploring the model with an alternative hill function**’). However, strictly speaking phase spaces should not be constructed in this way since the nullclines are not directly derived from the discontinuous gene regulatory model. Hence here, we employ the sigmoid function to avoid this problem and allow the full use of dynamical systems theory. However we note that the qualitative results obtained do not change if we use an alternative Hill-type Michaelis-Menten function (with Heaviside. See supporting information section ‘Exploring the model with an alternative Hill function’)

The shape of the function can be controlled by controlling the parameters θ and φ and sampling a space of these parameters allows the exploration of a large repertoire of potential regulatory relationships. We have plotted different versions of the sigmoid function with 4 different parameter sets to illustrate how these 2 parameters control the shape of the curve (Figure S3). θ is a quasi for cooperativity since it affects the steepness of the regulation curve (and is therefore the equivalent of the Hill coefficient in these types of functions). φ controls the sensitivity of the input-output relationship since it pushes the regulation curve to the right or to the left. These parameters have different biological interpretations depending on the exact components in our model as follows.

As mentioned in the main text, an S-shaped expression response is observed for LTR activation in cell lines following treatment with HDAC1 inhibitors strongly suggesting that cooperativity must be apparent at the LTR promoter when activated through acetylation (Archin et al., 2009). Though the exact cause of this cooperativity is currently unclear, one possibility is that the cooperativity stems from multiple acetylation of histone lysine residues each of which increasing the likelihood of another lysine being acetylated. Indeed, the histones of nucleosome 1 have been shown to be acetylated at multiple lysines during LTR induction suggested that this could be the case (Lusic et al., 2003). Therefore, for the HIV system θ represents biologically the degree of cooperativity in LTR activation that is suspected to be due to the multiple acetylation at nucleosome 1. φ controls the sensitivity of TAT expression to the balance of acetylase/deacetylase bound at nucleosome 1 and can therefore be thought to represent biologically the acetylation reaction rate of YY1/LSF.

Active caspase is commonly a tetramer of 2 small/large subunit dimers (Shi 2004). For the CAS and XIAP components therefore θ biologically represents the degree of cooperative oligomerisation of the caspases. Biologically φ by contrast represents the caspase cleavage reaction rate since it controls the sensitivity of caspase cleavage to the amount of substrate bound active caspase oligomer.

Saturation in the model stems from different features depending on the specific component. For Tat expression, the saturation could stem from the fact that there is a maximum rate at which RNAPII can transcribe the LTR in combination with the limited number (usually 1) of integrated HIV viruses. Once activation of the LTR promoter reaches a certain point therefore, it is impossible to generate Tat mRNAs at a higher frequency. Caspase and XIAP activation saturation stems from the limited size of the pro caspase and XIAP pools in a cell respectively. Again, once the caspases are activated beyond a certain level there is no procaspases (assuming the activation rate is beyond the expression rate of the procaspases) available to generate an activated caspase.


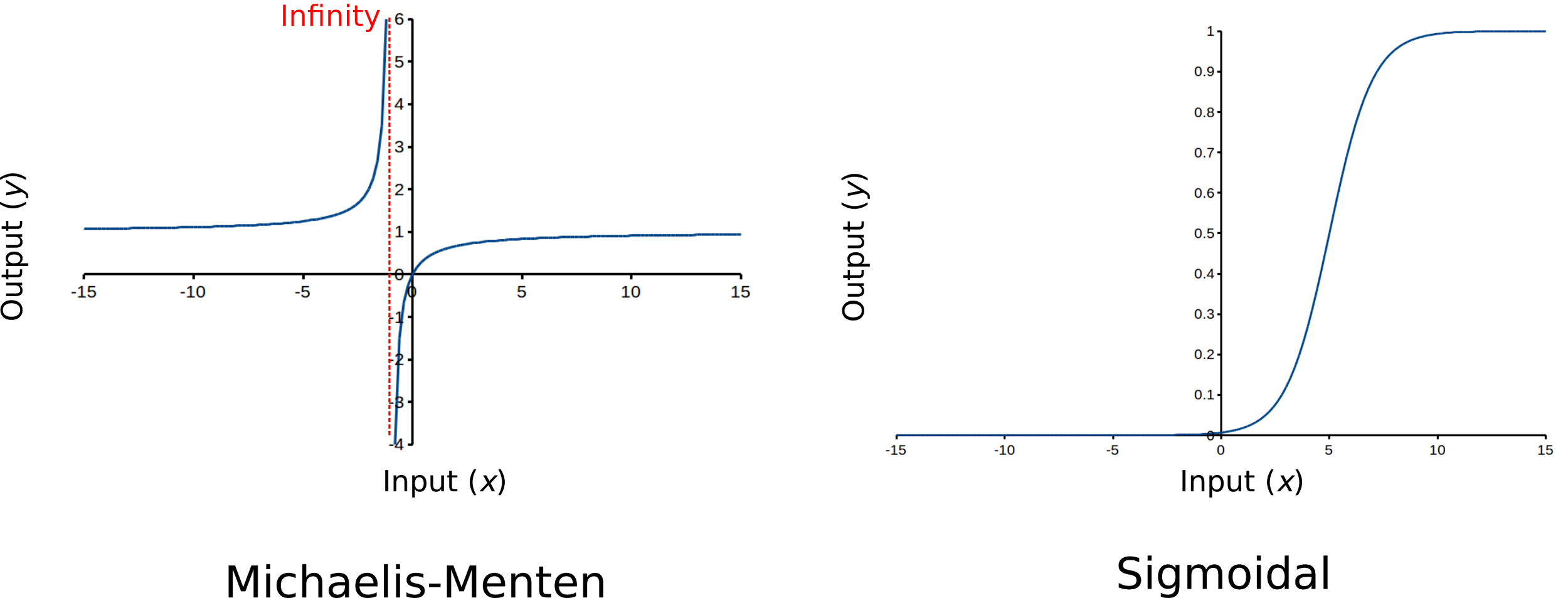


**Figure S1:** Demonstrating the input-output relationship of a Hill-type Michaelis Menten function (left; defined by equation 19) and Sigmoidal function (right). The sigmoidal function is continuous and always gives a positive output whilst the Michaelis Menten function can result in both negative and infinite output values. Parameters for sigmoidal function are θ=1 and φ=0.1.


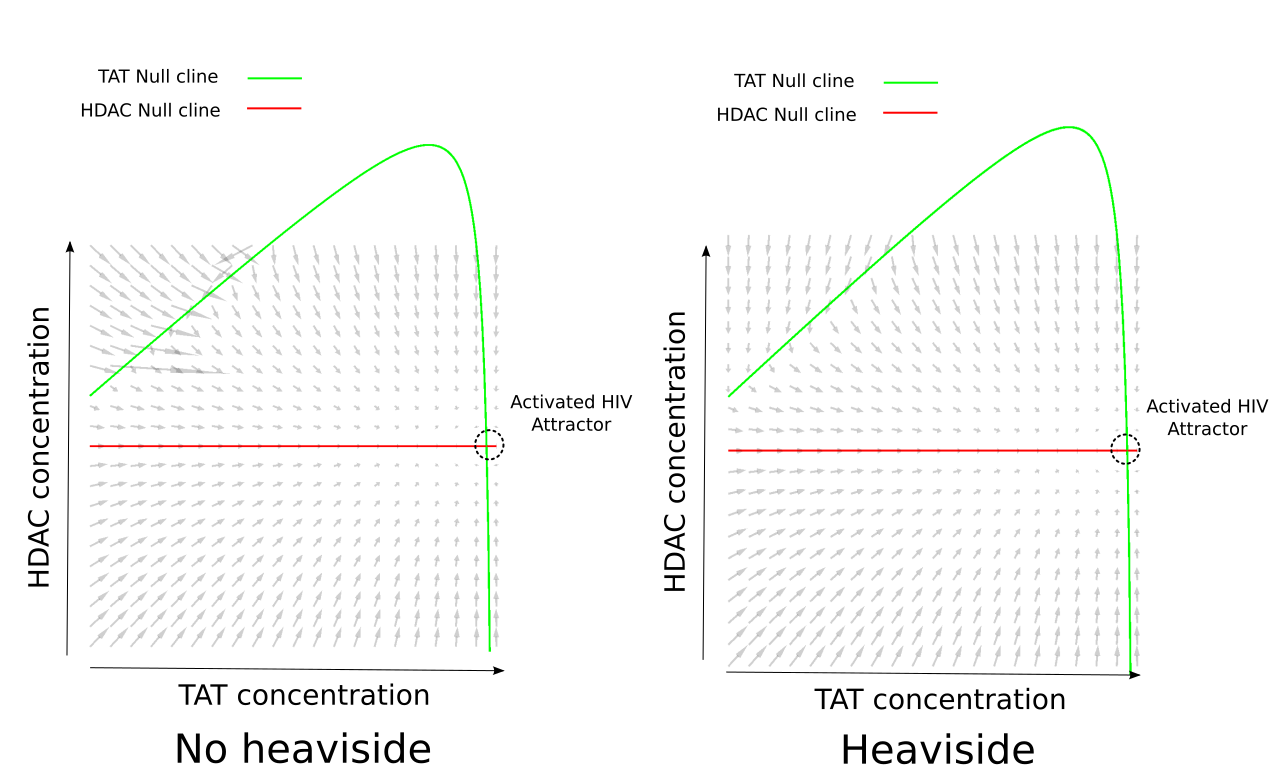


**Figure S2:** Phase spaces and nullclines built from a Michaelis-Menten gene regulatory model with and without a heaviside function. Equations 3 and 20 are used for this model. The Heaviside function is removed from equation 20 to build the phase space on the left. Parameters are α=1.8, β=2, ρ=25, γ_T_=0.05, γ_H_=0.05.


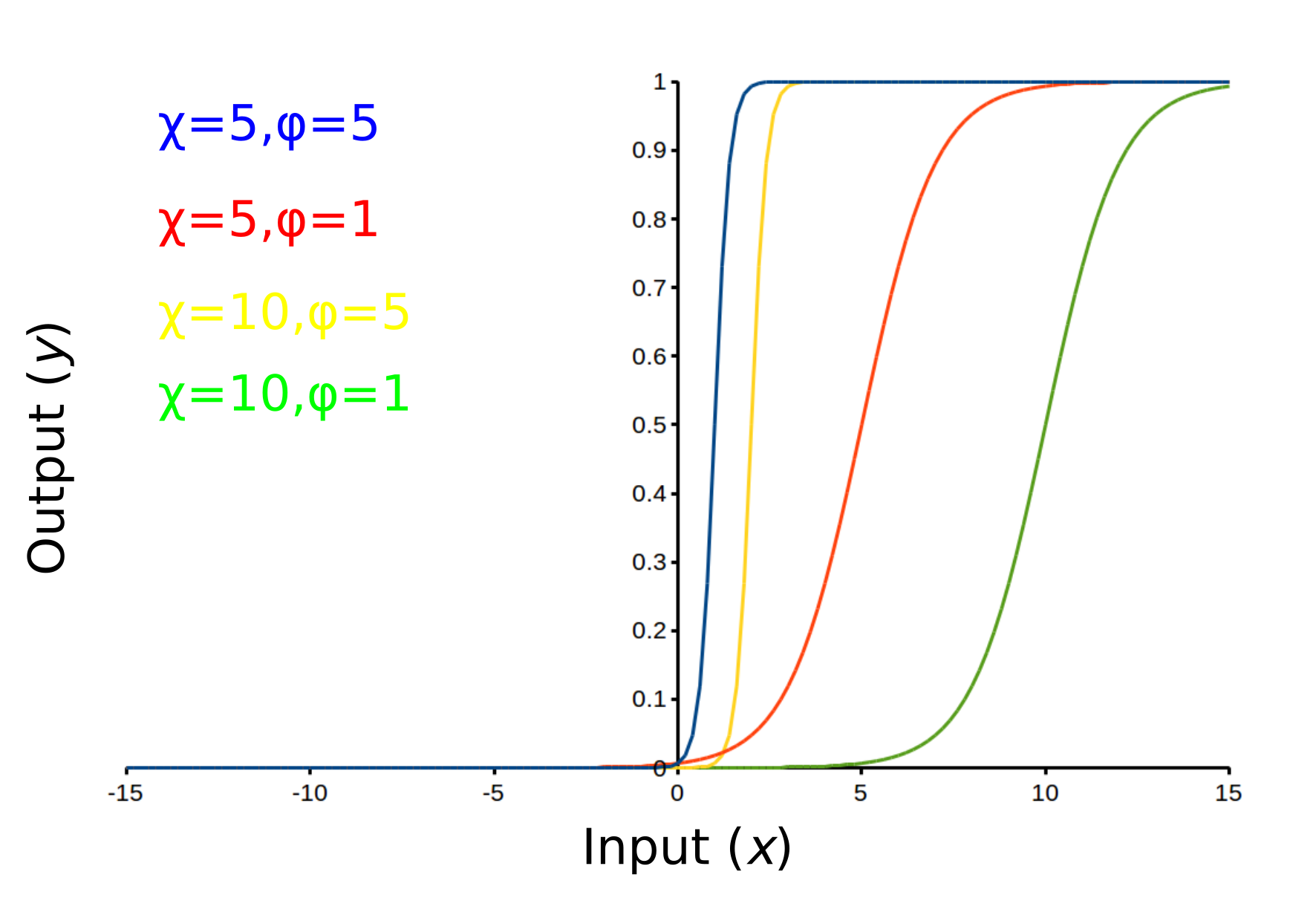


**Figure S3:** Examples of the varying shape of the sigmoid regulatory function with different parameter sets.

**Biological meaning of the parameters**

Due to the abstract nature of the model, the parameters do not always represent one specific feature but instead can capture a group of features. α, β, ε, η and ξ are the strength of regulation of components on one another which encompass multiple binding affinities as follows:

1. TAT self-activation strength (α): represents a combination of the binding affinity of TAT for p300 and the TAR element.
2. HDAC1 inhibition of TAT strength (β): the parameter represents a combination of HDAC1 binding affinity for YY1 and LSF.
3. CAS3/7 self-activation strength (ε): represents a combination of the binding affinities of CAS3/7 for CAS6, CAS6 for CAS8 and CAS8 for CAS3/7.
4. XIAP inhibition of CAS3/7 strength (η): represents the binding affinity of XIAP for the active sites of Cas3 and 7 through its BIR domains.
5. CAS3/7 inhibition of XIAP strength (ξ): represents the binding affinity of Caspase 3 for XIAP.

Note that the units and sampling rates are described in the section ‘Parameters and Units’. These binding affinity parameters thus define the balance of bound histone acetylase and deacetylase at nucleosome 1 in the HIV scenario (a positive value meaning there is net acetylase bound and negative value meaning net deacetylase bound) and the quantity of active CAS tetramer bound to its substrate in the apoptosis scenario. The sensitivity parameter φ then can be thought of as acetylation reaction rate of YY1/LSF in the TAT scenario and the average cleavage reaction rate of Cas3/6/7/8 in the CAS scenario. Cooperativity (the level of which defined by θ) due to multi-acetylation at nucleosome 1 and tetramerization of the caspases is then proposed to transform this linear relationship with saturation into a sigmoidal relationship. θ and φ thus control the shape of the regulatory input-output curve and are described in more detail in the previous section ‘Gene regulatory function’.

**Parameters and Units**

**Drug dosage and potency**

Typical doses of drug used in clinical settings span a wide range of concentrations consisting of several orders of magnitude (<1ug/kg to >1g/kg). Furthermore molecular weights of drugs vary from approximately 100g/mole for small molecules to 100,000g/mol for a large protein. Resulting molarity of drug therefore also spans over several orders of magnitude depending on the specific drug. Assuming a volume of distribution of 5 litres, this equates to a wide dynamic range of drug running from 1.4x10^-10^M (assuming 100,000g/mol at 1ug/kg concentration in a 70kg body in 5 litres) to 0.14M (assuming 100g/mol at 1g/kg concentration in a 70kg body in 5 litres) spanning 9 orders of magnitude. We therefore used a drug dose range that runs from 0.1nM to 0.1M in this study and state variables are measured in concentration (Molar).

We employed a drug potency bias (ρ>>ω) to select for drugs that had a therapeutic window activating the HIV system more than the apoptosis system. Specifically we randomly selected values of drug potency in the ranges 0 to 1x10^2^ for ρ and 0 to 1x10^-3^ for ω. ρ and ω are dimensionless.

**Time, half-life/decay parameters and their sampling**

We set our system for one iteration to represent 1 hour of real time and we measured in hours. Our drug decay parameter (*μ*) ranges from 0 to 0.99 which corresponds to a minimal drug half life (*κ*) of 0 minutes and a maximum drug half life of 69 hours ((0.99^69)≈0.5) since the drug concentration after a single application is defined by the following function

$D_{t}=D_{0}\mu^{t}$, (8)

where *D_t_* is the drug concentration at time t (discrete iterations) and *D_0_* is the initial drug concentration (see equation 5 in main manuscript for full definition of drug dose at any one time point when multiple drug applications are used). This places our drug half lives within a suitable physiological range since drug half lives are known to vary widely with drugs such as cytokines having half lives shorter than 20 minutes whilst drugs such as cyclosporin having half lives up to 19 hours. The drug decay parameter (*μ*) is sampled uniformly through the range 0 to 0.99 which maps to a negative exponential distribution of half-lives (since κ=ln(0.5)/ln(μ)). Both the drug decay parameter (*μ*) and drug decay half life (*κ*) are dimensionless.

Our gene product decay parameter (*γ_T_*, *γ_H_*, *γ_C_* and *γ_X_*) ranges from 0 to 0.1 which corresponds to a maximum gene product half life of infinity and a minimum half life of 6.5 hours in our system (((1-0.1)^6.5)≈0.5) since the gene product concentration is time stepped according to the following equation when there is no expression

$T=T_{0}\left( 1-\gamma_{T} \right)^{t}$*,* (9)

where $T_{t}$is the TAT concentration at time t, $T_{0}$ is the initial gene product concentration and *γ_T_* is the gene product decay parameter of TAT. TAT (*T*) in this case can be replaced with any of the other variables (either *C, H* or *X*) of the system in this equation along with their respective decay parameters (*γ_C_*, *γ_H_* and *γ_X_*). The average half life in our system when averaging over the sampled negative exponential range is approximately 25 hours. A large scale survey of mammalian protein half lives has uncovered a median of approximately 46 hours with the vast majority of proteins having a half life greater than a few hours (Schwanhäusser et al., 2011). Therefore decay parameters resulting from our random screen are placed within a suitable physiological range. The gene product decay parameter is sampled uniformly through the range 0 to 0.1 which maps to a negative exponential distribution of half-lives (since *λ*=ln(0.5)/ln(1-*γ*)). Both the gene product decay (*γ_T_*, *γ_H_*, *γ_C_* and *γ_X_*) and the gene product half life (*λ*) parameters have units measured in t^-1^ where t is time.

**Sampling of other parameters**

All parameters are sampled randomly through a exponentially decaying distribution to bias small numbers. For all parameters except the drug decay (*μ*) and gene product decay (*γ_T_*, *γ_H_*, *γ_C_* and *γ_X_*) parameters (see above) these values are sampled using the function

$v=Me^{-z}$, (10)

where *v* is the randomly chosen value for the parameter, *M* is the maximum value of the parameter range and *z* is a randomly chosen floating point number between 0 and 5. The drug decay (*μ*) and gene product decay (*γ*) parameters are sampled uniformly through their respective ranges since they are already converted to an exponentially decaying distribution when they are mapped to their respective half lives (*κ* and *λ* respectively). The form of the parameters *μ* and *γ* simply serve as a convenient way to encode the half lives in a discrete time stepping program that simulates the gene regulatory networks.

The input scalar (*θ)* and universal scalar (*φ)* parameters control the shape of the regulatory function and are dimensionless. K_M_ represents the maximum production rate of a particular component of the system and therefore has units of ct^-1^ (where c is concentration and t is time). The regulation parameters *α, β, ε*, *ρ, η* and *ω* have units c^-1^ where c is gene product concentration. These units maintain the equations dimensionless. We set the regulation and scalar parameter sampling ranges to allow for screening of the full dynamic range of the sigmoid input-output function. Specifically the regulation parameter range was set to have a maximum limit of 1 and the input scalar (*θ*) and universal scalar (*φ*) parameter ranges were set to have a maximum limit of 10. When we explore TAT and CAS activation using dosages through this sampled range responses are found that range from 0 to saturated over the drug input range or anything in between and with variable steepness. We are thus exploring a vast dynamic range of the input-output function and therefore it is likely that we are sampling the full range of mechanistic possibilities.

**Simulation initial conditions:**

Concentrations of TAT and Cas were set to 0 at the start of the simulation. Concentrations of HDAC1 and XIAP were set to 1nM Molar. Drug initial concentration was as indicated by the dose and the concentration changes as described by equations 5 and 6 in the main text.

**Null cline equations and phase spaces:**

We derived the following set of null cline equations to describe the HIV and apoptosis systems

TAT nullcline

$H=\left( \frac{\left( \left( \frac{\left( \varphi-\left( log\left( \left( \frac{K_{M}}{\gamma_{T}T} \right)-1 \right) \right) \right)}{\theta} \right)-\left( \rho+\alpha T \right) \right)}{-\beta} \right)$ (11)

CAS nullcline

$X=\left( \frac{\left( \left( \frac{\left( \varphi-\left( log\left( \left( \frac{K_{M}}{\gamma_{C}C} \right)-1 \right) \right) \right)}{\theta} \right)-\left( \omega+\varepsilon C \right) \right)}{-\eta} \right)$ (12)

HDAC nullcline:

$H=\frac{K_{M}}{2\gamma_{H}}$, (13)

XIAP Nullcline:

$X=\frac{K_{M}}{\gamma_{X}\left( 1+e^{\left( \varphi-\theta\left( -\xi C \right) \right)} \right)}$, (14)

Phase spaces were generated by simulating the change of concentration of the gene productions at each concentration combination of TAT/CAS and HDAC/XIAP for a single iteration. We simulated the model at 20 increments of 1 Molar. Maximum concentration of gene products is 20 Molar because the gene product decay parameter is set to 0.05t^-1^ for all components in the plotted parameters sets (see below). Maximum expression of a promoter is 1 Molar and hence maximum concentration occurs when maximum expression balances decay at 1/0.05=20Molar. Grey arrows on the phase space are plotted with their start coordinate at the concentration combination tested and their end coordinate at the resulting concentration after an iteration of the simulation.

Specific parameter sets used in the nullcline equations to make the figure 2 panels are as follows.

2e: α=1.8, β=5, ρ=0, φ=5, θ=5, γ_T_=0.05, γ_H_=0.05

2f: α=1.8, β=2, ρ=0, φ=5, θ=5, γ_T_=0.05, γ_H_=0.05

2g: α=5, β=0.05, ρ=0, φ=5, θ=5, γ_T_=0.05, γ_H_=0.05

2h: ε=0.3, η=2, ω=0, φ=0.001, θ=5, ξ=0.015, γ_C_=0.05, γ_X_=0.05

2i: ε=1.8, η=2, ω=0, φ=0.001, θ=5, ξ=0.1, γ_C_=0.05, γ_X_=0.05

2j:ε=1, η=0.1, ω=0, φ=0.001, θ=5, ξ=0.1, γ_C_=0.05, γ_X_=0.05

2k left: α=1.8, β=2, ρ=25, φ=5, θ=5, γ_T_=0.05, γ_H_=0.05

2k right: ε=1.8, η=2, ω=10, φ=0.001, θ=5, ξ=0.1, γ_C_=0.05, γ_X_=0.05

**Stability analysis:**

We wished to explore the stability of the fixed points within the system and how generally these results hold for different parameter sets. Hence we performed stability analysis for both the HIV reactivation and Apoptosis systems. We could reduce both the HIV reactivation system and apoptosis systems to a single dimension when considering equilibrium by deriving the following equation from equation (3) for HDAC

$H=\frac{1}{\lambda_{H}}$, (15)

And deriving the following equation from equation (4) for XIAP

$X=\frac{1}{\lambda_{X}\left( 1+e^{\left( \varphi-\theta\left( -\xi C \right) \right)} \right)}$, (16)

By feeding these equations into equations (1) and (2) we can derive the following equations that describe the system in terms of TAT or Cas concentrations alone:

$\frac{dT}{dt}=\frac{1}{\left( 1+exp\left( \varphi-\theta\left( \rho+\alpha T-\frac{\beta}{\lambda_{H}} \right) \right) \right)}-\lambda_{T}T$, (17)

$\frac{dC}{dt}=\frac{1}{\left( 1+e^{\left( \varphi-\theta\left( \omega+\epsilon C-\frac{\eta}{\lambda_{X}\left( 1+e^{\left( \varphi-\theta\left( -\xi C \right) \right)} \right)} \right) \right)} \right)}-\lambda_{C}C$, (18)

By plotting the flux components (synthesis and decay, the left and right hand terms of the above equations respectively) as a function of TAT or CAS concentration one can determine the stability of the fixed points. An example for TAT is shown in figure S1 but the shape/configurations are similar for CAS. Three possible configurations of these flux distributions are possible (Figure S4a-c). When decay is higher than synthesis TAT concentration reduces as demonstrated by a left-facing arrow on the stability line below. When synthesis is higher than decay TAT concentration increases as demonstrated by a right-facing arrow on the stability line. The stability analysis demonstrates that for any cell the low fixed point (HIV inactive) and high fixed point (HIV reactivated, if it exists) are always stable (irrespective of flux configuration). The third fixed point, is always unstable when it exists. The same is thus true for the Apoptosis system with the apoptosis and live cell fixed points being stable and the intermediate fixed point unstable when it exists (Figure S4d-f).


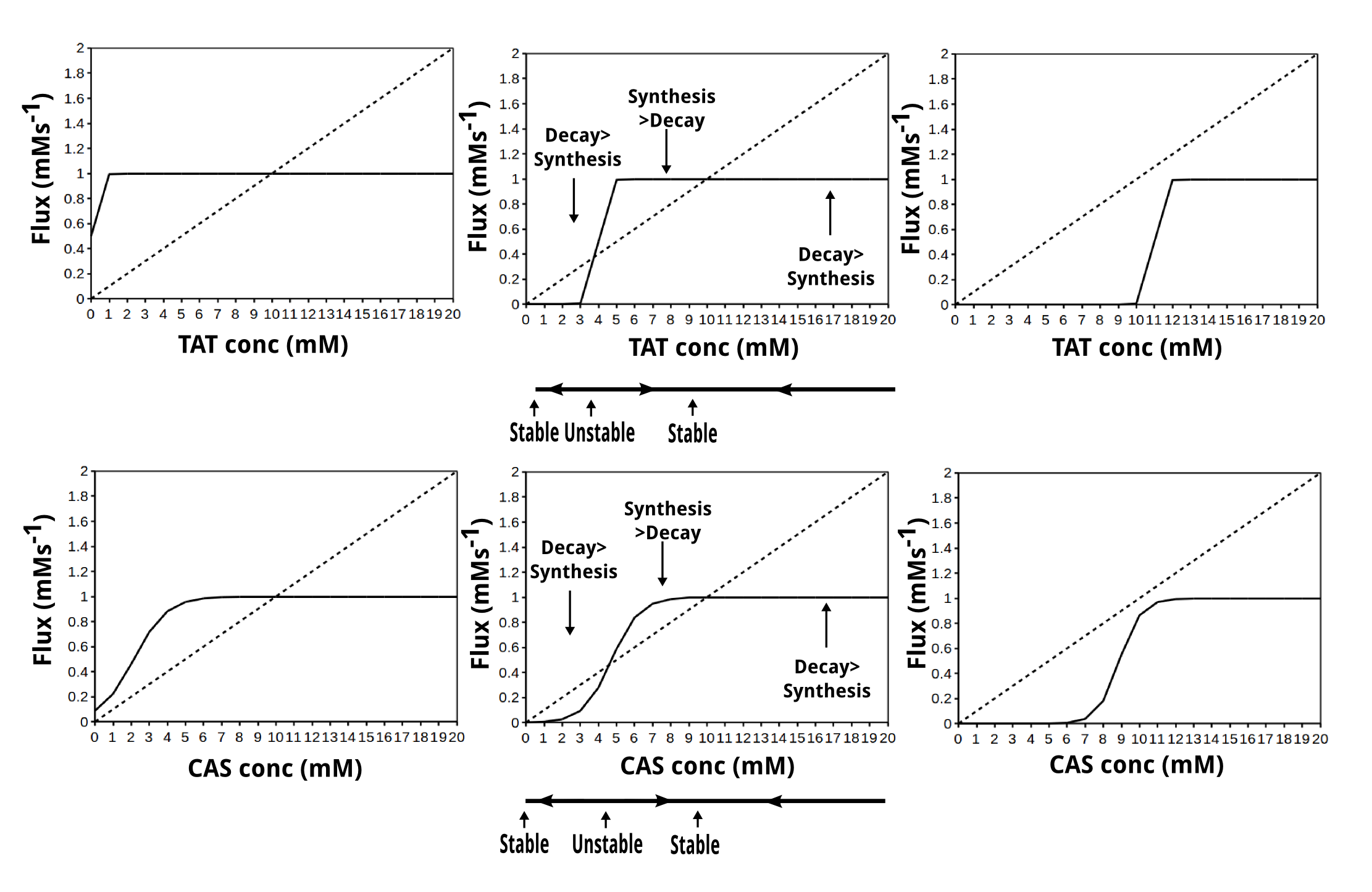


**Figure S4:** Stability analysis demonstrates the possible configurations of each system. The HIV reactivation system is shown above and the Apoptosis system below. Gene product synthesis is shown by the unbroken line and gene product decay shown by the dashed line. Regions where synthesis is greater than decay and vice versa are indicated. Fixed points in the system occur where the two lines intersect. The stability of those fixed points can be determined by the net flux either side as demonstrated by the thick line underneath. Arrowheads indicate the direction of the system. The parameters used for the reactivation system are *θ=5, φ=5,* α=1, γ_H_=0.05, γ_T_=0.1, ρ=2 , β=0.05 on the left, β=0.25 in the middle and β=0.6 on the right. The parameters used for the apoptosis system are *θ=1, φ=1,* ε=1, γ_X_=0.01, γ_C_=0.1, ω=0.01, κ=0.1, η=0.05 on the left, η=0.2 in the middle, η=0.6 on the right. Decay is set at 0.1 in all cases.

**Comparing the desired-effect/side-effect balance with different frequencies of interval dosages**

In a realistic drug application setting it is often not pragmatic nor desirable to use continuous drug delivery. More commonly the aim is to alter the frequency of drug application to improve the balance. Hence we explored the difference in desired-effect/side-effect trade-off for drugs delivered at interval doses but at different frequencies (with the same drug exposure, AUC). Hence for each of the 100 hypothetical drugs in our screen we calculated a random AUC time-dose value by randomly choosing a dose between the MED and MTD, a random number of bouts between 1 and 10 and a random time per bout. We then simulated each hypothetical drug 10 times with between 1 and 10 bouts of drug application over the same time period , adjusting the concentration of applied drug and time per bout accordingly to maintain the same simulation time frame (randomly chosen between 1 and 2000 hours) and the same drug exposure (AUC). We measured the desired cell state switching relative to the side-effect cell state switching for each different interval frequency for each different drug. The results are shown in figure S5 and demonstrate that better desired-effect/side-effect balance is achieved as drugs are applied more frequently but with a lower dose such that the overall AUC is identical between the tests. There is a strong positive linear correlation between interval frequency and desired-effect/side-effect balance.


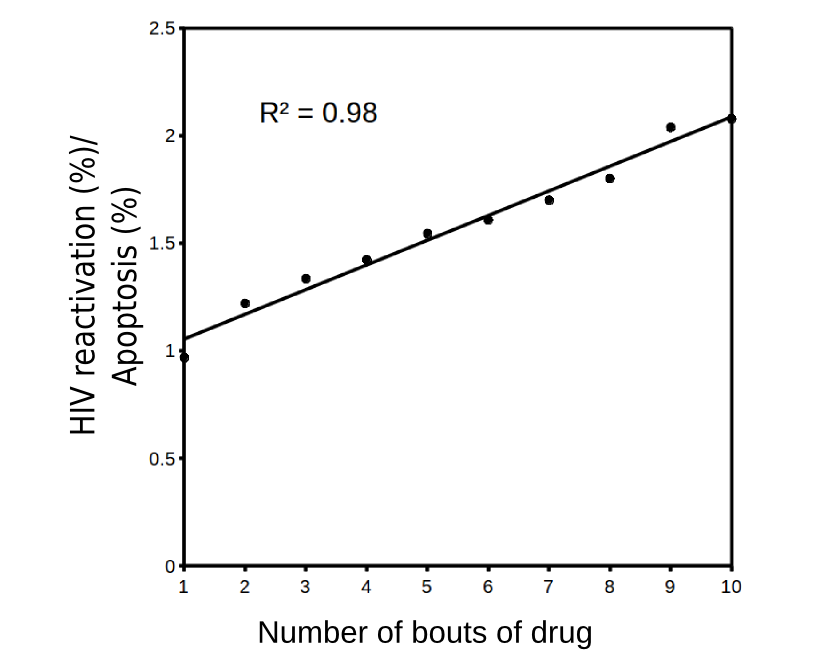


**Figure S5: The desired-effect/side-effect balance improves with increasing frequency of interval dosages at the same drug exposure.** We measured desired effect relative to side-effect (*y*-axis) for different frequencies of drug application (*x*-axis - number of bouts). Each point represents the average of 100 hypothetical drugs. A positive linear trend line and corresponding R^2^ is shown.

**Exploring the effects of stochastic noise on the model**

Due to the typically single copy of LTR in a latently infected T cell, we expect there to be extreme stochasticity in expression from the LTR. Indeed, extreme stochasticity has been observed through time-lapse imaging of single cells infected with a single LTR (without the auto-positive TAT feedback loop) and a fluorescent readout (Razooky et al., 2011). Therefore, in order to explore the effects of stochastic noise, we implemented stochastic noise at the LTR in our model. We implemented noise by adding a noise term that changes the expression value of the TAT gene product at every iteration of the simulation. This noise term randomly increases or decreasing the amount of TAT produced by up to +/-5% or +/-25% per iteration. We explored whether this amount of noise is suitable by simulating our model over time with the auto-positive feedback of TAT>TAT removed. In this way, we could directly compare the expression levels in our model to those shown in Razooky et al., 2011 Figure 4a. We simulated 10 cells for 20 hours that started with identical TAT concentrations and explored how expression changed over time. The results are shown in Figure S6 and demonstrate that expression levels are observed that span up to an order of magnitude which is comparable to the diversity in TAT expression levels measured in Razooky et al. Therefore, these per iteration noise levels seems to be representative of the real level of stochasticity measured at the LTR.

We then performed our full set of assays using the +/-5% or +/-25% noise per iteration. The full results are shown in figures S7 and S8 demonstrating that the addition of stochastic noise at the LTR promoter has little effect on the actual qualitative result and in particular the main message that continuous dosing is superior to discontinuous dosing (The maximum HIV reactivation possible is however reduced for both the continuous and discontinuous regimens in the +/-25% per iteration example and the bout relationship supports a logarithmic trend better).

The fact that the results stay essentially the same suggests that stochastic noise does not impact on the conclusions from the dynamical systems theory analysis. However, to explore the relationship to the dynamical systems theory analysis further we created a phase space using the +/-5% stochastic model described above applied to the drug-applied nullcline setup of figure 2k (Figure S9 left). The nullclines per se are not changed since they simply define the concentrations of the system where there is no flux in one of the variables. However, the underlying phase spaces become ‘wobbly’ as would be expected with the addition of stochastic noise at the level of the LTR promoter. Note that the phase space only becomes wobbly in the x-axis as the direction of the arrows in this axis represents the change in TAT concentration (i.e. amount of expression).

What is clear from this scenario is that the stochastic noise cannot affect the result because the null clines do not move and therefore the basic set of attractors available to the system is

unchanged. Stochastic noise therefore may cause a cell to locally jump to a different position in phase space but that new position is still in the same basin of attraction. In more complex contexts where there are multiple attractors and basins of attraction this is not necessarily the case as stochasticity can cause cells to jump from one basin of attraction to another if they are sufficiently close to the separatrix. Here however as the simulation results have confirmed, the addition of stochastic noise does not affect the conclusions from the nullcline analysis.

An alternative way of envisaging the effect of stochastic noise on the phase spaces/nullclines is to plot the nullclines as a probability density function (illustrated by figure S9 right). This can be interpreted as the probability of coordinates on the phase space giving zero-flux. In this case the nullclines become ‘blurred’ and when they overlap such as when the drug dose is close to MEBD/MTBD (close to a bifurcation-see figure 6 centre) this affects the behaviour of the system. In this situation, a stable attractor (in this case the inactivated HIV attractor) would be expected to exist in a state of flux, appearing and disappearing stochastically depending on the amount of overlap in the nullclines (which in turn depends on the level of stochastic noise). However, the basic conclusion also does not change under this interpretation since once the TAT nullcline has been translated sufficiently, the effect of the overlap is negligible.

Note, each iteration in our model is abstract representing up to several hundred potential expression events from the single LTR promoter in each infected cell. Direct measurements of RNA biogenesis from the HIV-1 promoter using live cell imaging has been measured to be as fast as 50kb/min (Maiuri et al., 2011; Marcello 2012). The full HIV-1 locus is approximately 10kb long. Therefore, theoretically several new mRNA molecules can be produced every minute, or about 300 new mRNA molecules every hour. Hence changing the expression value of the LTR by +/-5% or +/-25% is analogous to stochastic chance that these expression events do or do not occur since as we have described previously each iteration of our simulation represents 1 hour of real time. Finally, it should also be noted that the stochastic noise we have used here does not have the autocorrelation that would be expected from the ‘bursty’ nature of gene expression at this locus (Dar et al., 2012). Exploring the effect of continuous/discontinuous dosing in a model that explicitly produces bursty gene expression such as that used by Weinberger and colleagues will thus yield deeper insight into the exact effect of stochastic noise.


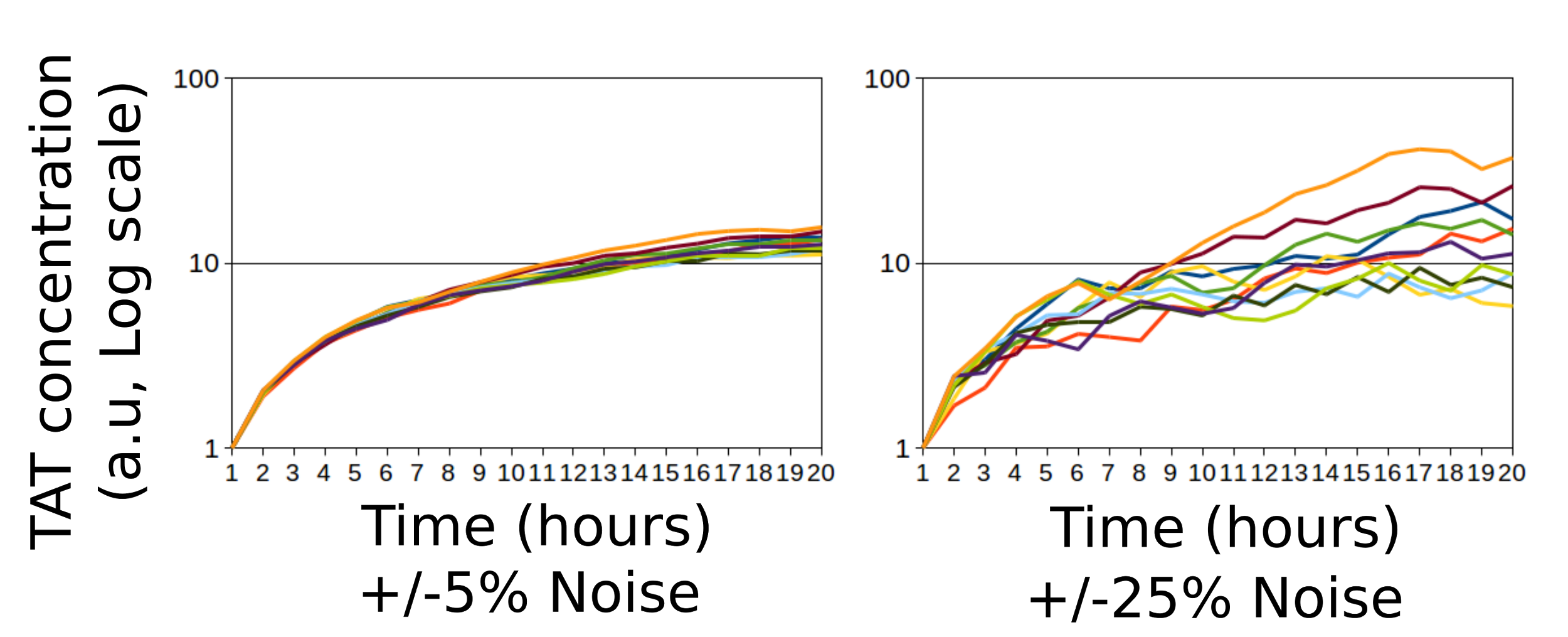


**Figure S6: Exploring the change in expression of TAT over time.** (left) With +/-5% noise and (right +/-25% noise. x-axis is time and y-axis is concentration (log scale). 10 different cells are shown (different coloured lines). Parameters used are α=1.8, β=2, ρ=25, φ=5, θ=5, γT=0.05, γH=0.05. All cells started from a concentration of 0.1 for TAT.


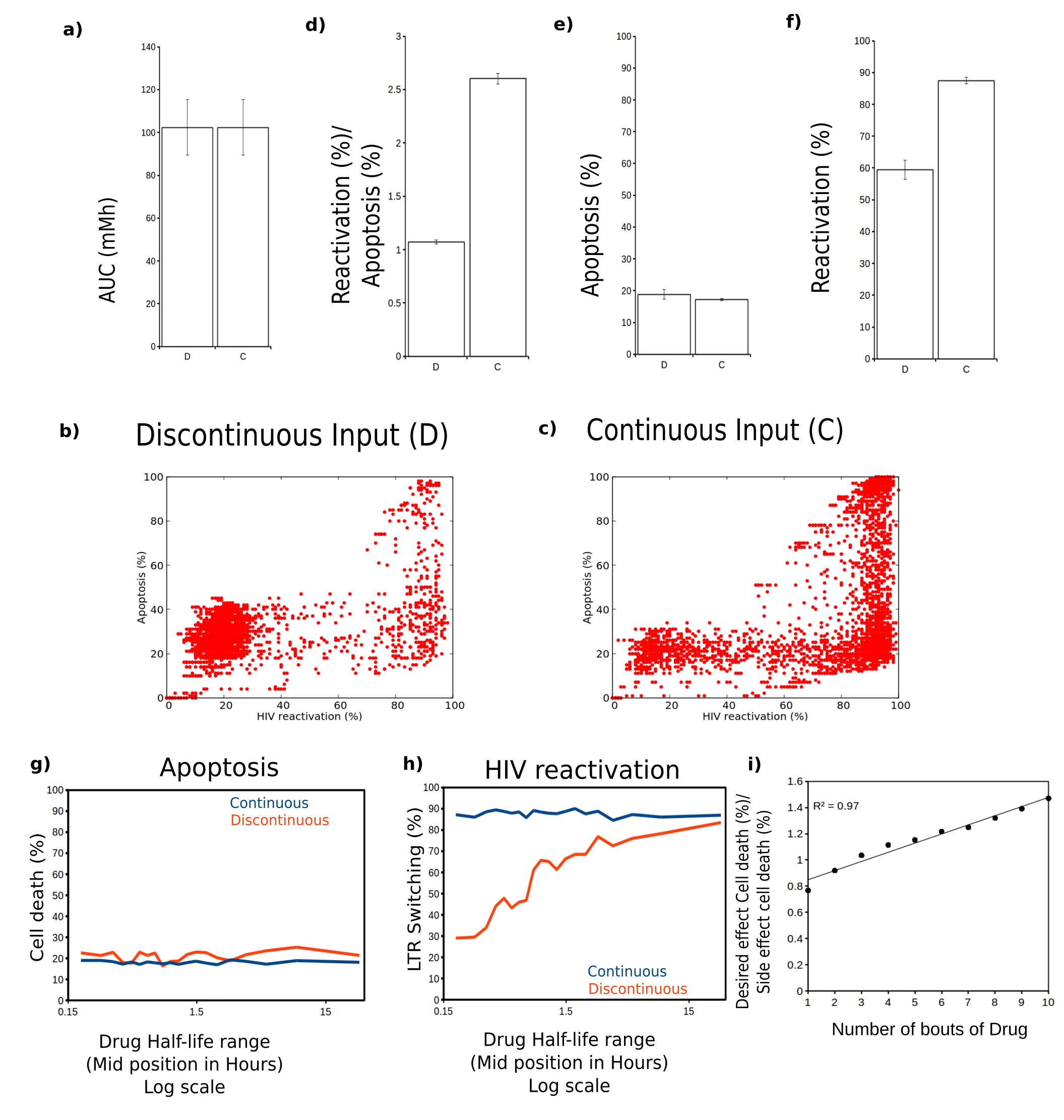


**Figure S7: The same qualitative result is obtained when simulating the model with stochastic noise up to +/-5% per iteration.** a) Measuring the total AUC (in milliMolar Hours) for the discontinuous and continuous regimens demonstrates that they are identical. (b and c) A scatter plot for reactivation frequency (x-axis) and apoptosis (y-axis) in the discontinuous (b) or continuous (c) regimen. Each red dot is one particular drug at one particular dose in the dosage range. d) Quantification of Switching relative to cell death for all points in b and c where cell death is not equal to 0. Standard error is shown. p<0.0001 that there is a difference between the two groups using a two tailed unpaired *t-*test. e) Quantity of apoptosis for the two regimens when we identify the best (maximal reactivation frequency with apoptosis below 20%) dose for each drug. The average apoptosis frequency for each drug is plotted on the y-axis. f) Quantity of reactivation for the two regimens when we identify the best (maximal reactivation frequency with apoptosis below 20%) dose for each drug. The reactivation frequency for each drug is plotted on the y-axis. p<0.0001 that there is a difference between the two groups using a two tailed unpaired *t-*test. (g and h) Reactivation and apoptosis frequency after screening for drugs with half lives in different ranges. g) Apoptosis frequency (y-axis) in different half life ranges (x-axis defines the midpoint of the sampled range). The continuous regimen is shown by the blue line and discontinuous regimen by the red line. h) Reactivation frequency (y-axis) in different half life ranges (x-axis defines the midpoint of the sampled range). The continuous regimen is shown by the blue line and discontinuous regimen by the red line. i) Desired effect relative to side-effect (*y*-axis) for different frequencies of drug application (*x*-axis - number of bouts). Each point represents the average of >100 hypothetical drugs. A positive linear trend line and corresponding R^2^ is shown.


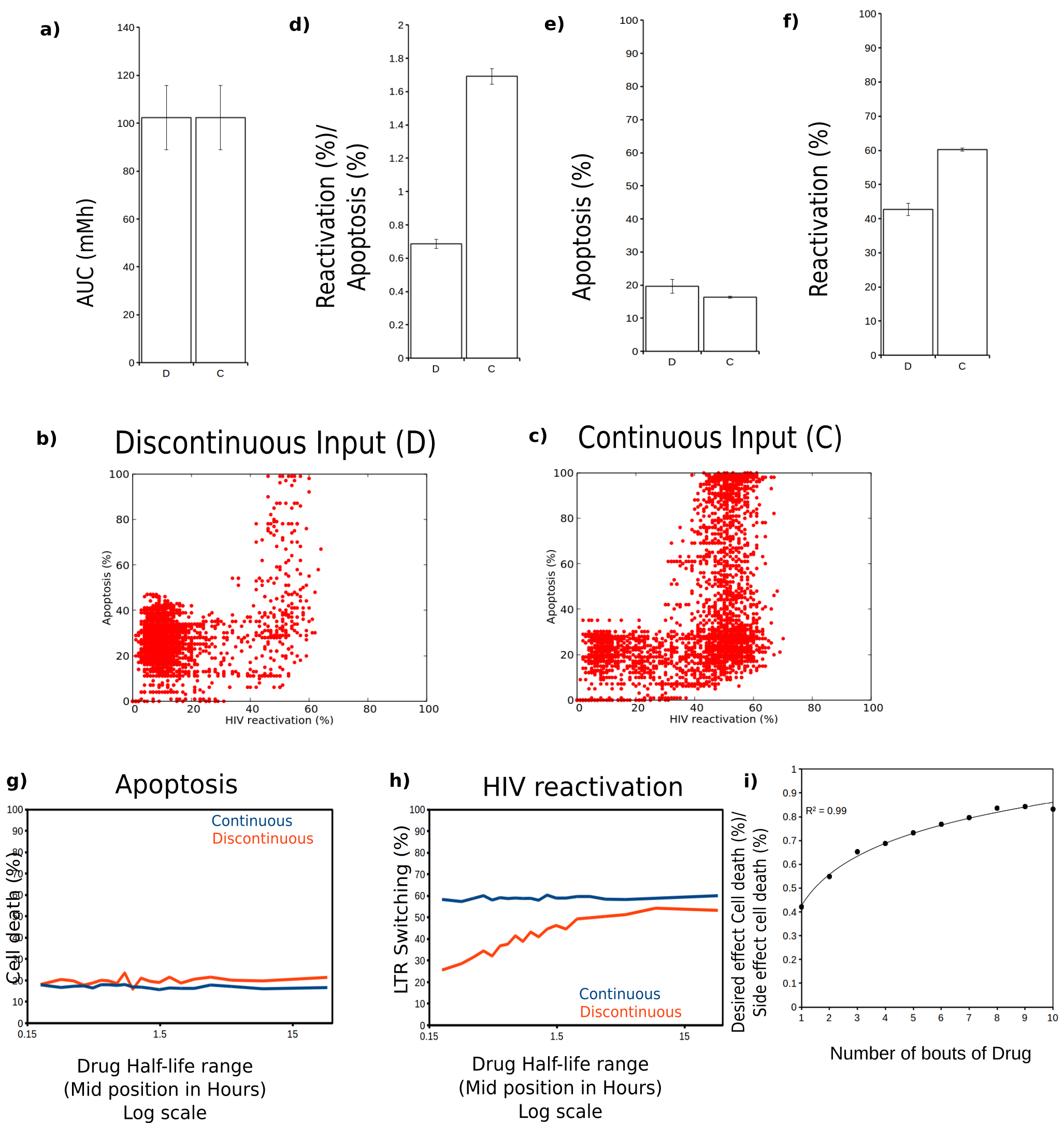


**Figure S8: A similar qualitative result is obtained when simulating the model with stochastic noise up to +/-25% per iteration.** a) Measuring the total AUC (in milliMolar Hours) for the discontinuous and continuous regimens demonstrates that they are identical. (b and c) A scatter plot for reactivation frequency (x-axis) and apoptosis (y-axis) in the discontinuous (b) or continuous (c) regimen. Each red dot is one particular drug at one particular dose in the dosage range. d) Quantification of Switching relative to cell death for all points in b and c where cell death is not equal to 0. Standard error is shown. p<0.0001 that there is a difference between the two groups using a two tailed unpaired *t-*test. e) Quantity of apoptosis for the two regimens when we identify the best (maximal reactivation frequency with apoptosis below 20%) dose for each drug. The average apoptosis frequency for each drug is plotted on the y-axis. f) Quantity of reactivation for the two regimens when we identify the best (maximal reactivation frequency with apoptosis below 20%) dose for each drug. The reactivation frequency for each drug is plotted on the y-axis. p<0.0001 that there is a difference between the two groups using a two tailed unpaired *t-*test. (g and h) Reactivation and apoptosis frequency after screening for drugs with half lives in different ranges. g) Apoptosis frequency (y-axis) in different half life ranges (x-axis defines the midpoint of the sampled range). The continuous regimen is shown by the blue line and discontinuous regimen by the red line. h) Reactivation frequency (y-axis) in different half life ranges (x-axis defines the midpoint of the sampled range). The continuous regimen is shown by the blue line and discontinuous regimen by the red line. i) Desired effect relative to side-effect (*y*-axis) for different frequencies of drug application (*x*-axis - number of bouts). Each point represents the average of >100 hypothetical drugs. A logarithmic trend line and corresponding R^2^ is shown.


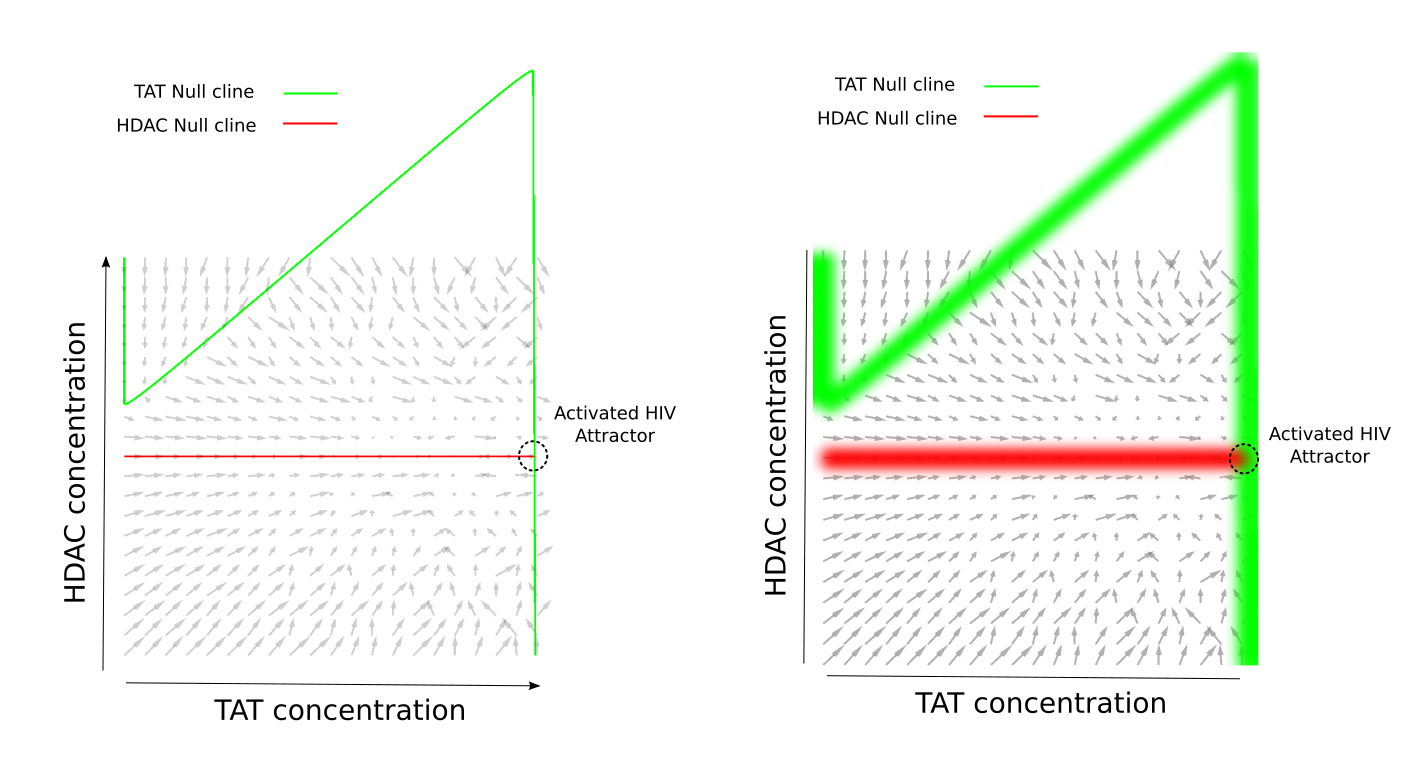


**Figure S9:** (left) Phase space shown in figure 2k but with +/-5% stochastic noise in expression from the LTR. (right) The same phase space but with probability density nullclines illustrated. Colour intensity corresponds to the probability of zero-flux at that set of variable concentrations.

**Implementing the drug as an inhibitor of HDAC instead of an activator of TAT**

Many drugs that have been utilized to attempt reactivation of latent HIV have focused on inhibiting HDAC. To explore whether our model can mimic the application of such drugs and to ascertain whether the results of our model are robust to changes in the way a drug affects the HIV system, we implemented the drug as an inhibitor of HDAC instead of an activator of TAT. The results are shown in figure S9 and demonstrate that the results are qualitatively the same for inhibiting HDAC instead of activating TAT. The effect is much weaker in this case though. Furthermore we increased the drug potency bias (ρ>>ω) in order to see a significant effect (Specifically we used ranges 1x10^6^ for ρ and 1x10^-6^ for ω; units are c^-1^ where c is drug concentration). This weakened effect and requirement for an increased parameter bias likely represents the abstract nature of our model rather than suggesting that activators of TAT are superior to inhibitors of HDAC. Many features such as explicit delays are not included in our model and would have important influences on the quantitative dynamics of the system.


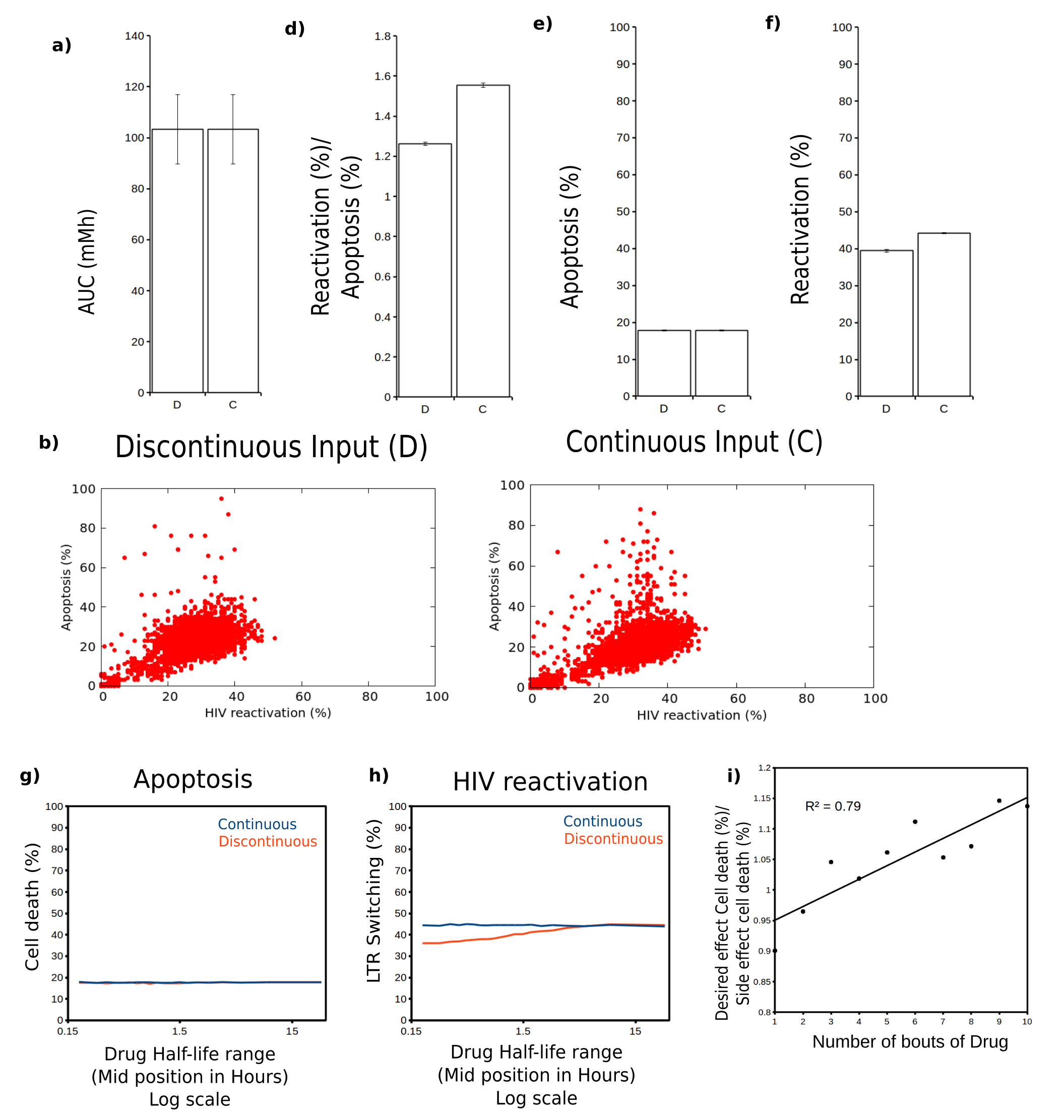


**Figure S10: The same qualitative result is obtained when selecting for drugs that inhibit HDAC instead of activating TAT.** a) Measuring the total AUC (in milliMolar Hours) for the discontinuous and continuous regimens demonstrates that they are identical. (b and c) A scatter plot for reactivation frequency (x-axis) and apoptosis (y-axis) in the discontinuous (b) or continuous (c) regimen. Each red dot is one particular drug at one particular dose in the dosage range. d) Quantification of Switching relative to cell death for all points in b and c where cell death is not equal to 0. Standard error is shown. p<0.0001 that there is a difference between the two groups using a two tailed unpaired *t-*test. e) Quantity of apoptosis for the two regimens when we identify the best (maximal reactivation frequency with apoptosis below 20%) dose for each drug. The average apoptosis frequency for each drug is plotted on the y-axis. f) Quantity of reactivation for the two regimens when we identify the best (maximal reactivation frequency with apoptosis below 20%) dose for each drug. The reactivation frequency for each drug is plotted on the y-axis. p<0.0001 that there is a difference between the two groups using a two tailed unpaired *t-*test. (g and h) Reactivation and apoptosis frequency after screening for drugs with half lives in different ranges. g) Apoptosis frequency (y-axis) in different half life ranges (x-axis defines the midpoint of the sampled range). The continuous regimen is shown by the blue line and discontinuous regimen by the red line. h) Reactivation frequency (y-axis) in different half life ranges (x-axis defines the midpoint of the sampled range). The continuous regimen is shown by the blue line and discontinuous regimen by the red line. i) Desired effect relative to side-effect (*y*-axis) for different frequencies of drug application (*x*-axis - number of bouts). Each point represents the average of >100 hypothetical drugs. A positive linear trend line and corresponding R^2^ is shown.

**Exploring the model with an alternative hill function**

We implemented an alternative Michaelis Menten (MM) function (which is a special case of the Hill function where the Hill coefficient is equal to 1). The MM function is described by

$O=\frac{I}{(1+I)}$, (19)

where *O* is the output and the input (*I*) equals the sum of the regulating genes multiplied by their respective concentrations (For example εC-ηX for Cas in the example without drug application example - figure 2a). Note we added a Heaviside function ζ*(x)* to prevent negative values resulting from the MM function such that ζ*(x)=1* if *x>=0* or ζ*(x)=0* if *x<0*. The full Hill equations are

$\frac{dT}{dt}=\zeta\left( \frac{\rho+\varepsilon T-\eta H}{(1+\rho+\varepsilon T-\eta H)} \right)\frac{\rho+\varepsilon T-\eta H}{(1+\rho+\varepsilon T-\eta H)}-\gamma_{T}T$, (20)

$\frac{dC}{dt}=\zeta\left( \frac{\omega+\varepsilon C-\eta X}{(1+\omega+\varepsilon C-\eta X)} \right)\frac{\omega+\varepsilon C-\eta X}{(1+\omega+\varepsilon C-\eta X)}-\gamma_{C}C$, (21)

$\frac{dX}{dt}=\zeta\left( \frac{-\xi C}{\left( 1-\xi C \right)} \right)\frac{-\xi C}{\left( 1-\xi C \right)}-\gamma_{X}X$. (22)

Note that the equation for HDAC does not change (equation 3 in main text). We sampled exactly the same parameter range as for the sigmoidal function. We only selected 100 hypothetical drugs as for the sigmoidal function. We explored the same set of model features as for the sigmoid function in exactly the same way. The results are shown in figure S11 and demonstrate that the qualitative result is the same for the MM as the exponential function, that continuous drug delivery at the same AUC allows a better desired-effect/side-effect trade-off compared to discontinuous drug delivery.

**
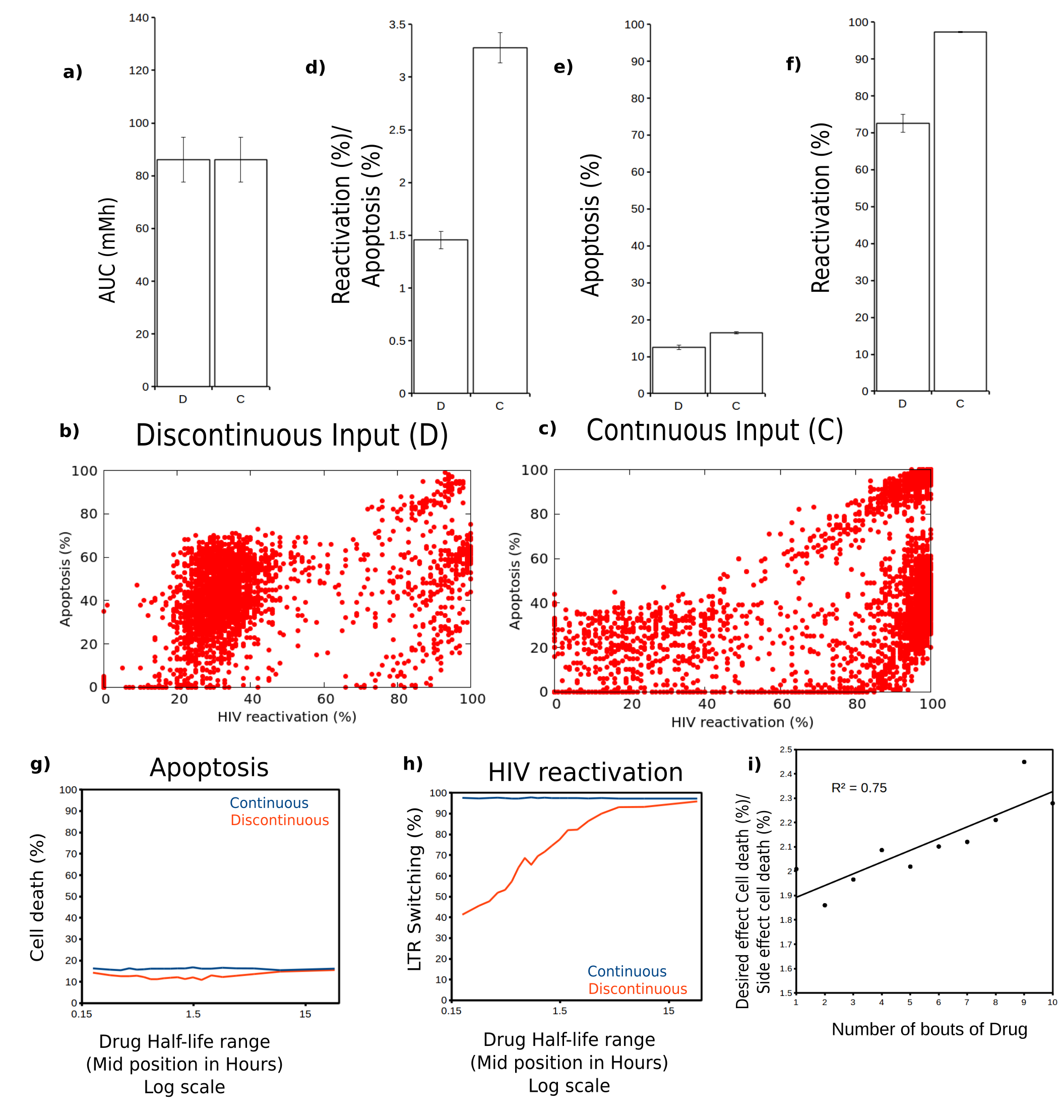
**

**Figure S11: The same qualitative result is obtained when using a Hill-type regulatory function.** a) Measuring the total AUC (in milliMolar Hours) for the discontinuous and continuous regimens demonstrates that they are identical. (b and c) A scatter plot for reactivation frequency (x-axis) and apoptosis (y-axis) in the discontinuous (b) or continuous (c) regimen. Each red dot is one particular drug at one particular dose in the dosage range. d) Quantification of Switching relative to cell death for all points in b and c where cell death is not equal to 0. Standard error is shown. p<0.0001 that there is a difference between the two groups using a two tailed unpaired *t-*test. e) Quantity of apoptosis for the two regimens when we identify the best (maximal reactivation frequency with apoptosis below 20%) dose for each drug. The average apoptosis frequency for each drug is plotted on the y-axis. f) Quantity of reactivation for the two regimens when we identify the best (maximal reactivation frequency with apoptosis below 20%) dose for each drug. The reactivation frequency for each drug is plotted on the y-axis. p<0.0001 that there is a difference between the two groups using a two tailed unpaired *t-*test. (g and h) Reactivation and apoptosis frequency after screening for drugs with half lives in different ranges. g) Apoptosis frequency (y-axis) in different half life ranges (x-axis defines the midpoint of the sampled range). The continuous regimen is shown by the blue line and discontinuous regimen by the red line. h) Reactivation frequency (y-axis) in different half life ranges (x-axis defines the midpoint of the sampled range). The continuous regimen is shown by the blue line and discontinuous regimen by the red line. i) Desired effect relative to side-effect (*y*-axis) for different frequencies of drug application (*x*-axis - number of bouts). Each point represents the average of >100 hypothetical drugs. A positive linear trend line and corresponding R^2^ is shown.

**Supporting References:**

Dar RD, Razooky BS, Singh A, Trimeloni TV, McCollum JM, Cox CD, Simpson ML, Weinberger LS. (2012). Transcriptional burst frequency and burst size are equally modulated across the human genome. *Proc Natl Acad Sci U S A.* **109:** 17454-9.

Lusic, M., Marcello, A., Cereseto, A., Giacca, M. (2003). Regulation of HIV-1 gene expression by histone acetylation and factor recruitment at the LTR promoter. *The EMBO Journal*, **22:** 6550–6561.

Maiuri P, Knezevich A, De Marco A, Mazza D, Kula A, McNally JG, Marcello A. (2011). Fast transcription rates of RNA polymerase II in human cells. *EMBO Rep.* **12:** 1280-1285.

Marcello A. (2012). RNA polymerase II transcription on the fast lane. *Transcription*. **3:** 29-34.

Schwanhäusser B, Busse D, Li N, Dittmar G, Schuchhardt J, Wolf J, Chen W, Selbach M. (2011). Global quantification of mammalian gene expression control. *Nature.* **473**: 337-42.

Shi Y. (2004) Caspase activation: revisiting the induced proximity model. *Cell.* **117**: 855-8.
